# Genome‐wide occupancy of histone H3K27 methyltransferases CURLY LEAF and SWINGER in *Arabidopsis* seedlings

**DOI:** 10.1002/pld3.100

**Published:** 2019-01-31

**Authors:** Jie Shu, Chen Chen, Raj Kumar Thapa, Shaomin Bian, Vi Nguyen, Kangfu Yu, Ze‐Chun Yuan, Jun Liu, Susanne E. Kohalmi, Chenlong Li, Yuhai Cui

**Affiliations:** ^1^ London Research and Development Centre Agriculture and Agri‐Food Canada London Ontario Canada; ^2^ Department of Biology Western University London Ontario Canada; ^3^ College of Plant Science Jilin University Changchun China; ^4^ Harrow Research and Development Centre Agriculture and Agri‐Food Canada Harrow Ontario Canada; ^5^ Guangdong Academy of Agricultural Sciences Guangzhou China; ^6^ State Key Laboratory of Biocontrol and Guangdong Key Laboratory of Plant Resources School of Life Sciences Sun Yat‐sen University Guangzhou China

**Keywords:** *Arabidopsis*, *CURLY LEAF*, *SWINGER*, genome‐wide occupancy, H3K27me3, gene repression

## Abstract

The Polycomb Group (PcG) proteins form two protein complexes, PcG Repressive Complex 1 (PRC1) and PRC2, which are key epigenetic regulators in eukaryotes. PRC2 represses gene expression by catalyzing the trimethylation of histone H3 lysine 27 (H3K27me3). In *Arabidopsis* (*Arabidopsis thaliana*), CURLY LEAF (CLF) and SWINGER (SWN) are two major H3K27 methyltransferases and core components of PRC2, playing essential roles in plant growth and development. Despite their importance, genome‐wide binding profiles of CLF and SWN have not been determined and compared yet. In this study, we generated transgenic lines expressing GFP‐tagged CLF/SWN under their respective native promoters and used them for ChIP‐seq analyses to profile the genome‐wide distributions of CLF and SWN in *Arabidopsis* seedlings. We also profiled and compared the global H3K27me3 levels in wild‐type (WT) and PcG mutants (*clf*,* swn*, and *clf swn*). Our data show that CLF and SWN bind to almost the same set of genes, except that SWN has a few hundred more targets. Two short DNA sequences, the *GAGA*‐like and *Telo*‐box‐like motifs, were found enriched in the CLF and SWN binding regions. The H3K27me3 levels in *clf*, but not in *swn*, were markedly reduced compared with WT; and the mark was undetectable in the *clf swn* double mutant. Further, we profiled the transcriptomes in *clf*,* swn*, and *clf swn,* and compared that with WT. Thus this work provides a useful resource for the plant epigenetics community for dissecting the functions of PRC2 in plant growth and development.

## 1 INTRODUCTION

The Polycomb Group (PcG) proteins play crucial roles in epigenetic regulation through maintaining the repressed state of target genes (Dellino et al., 2004). Initially described in *Drosophila melanogaster* as regulators of gene expression (Lewis, 1978), PcG proteins form two functionally distinct multi‐protein complexes known as Polycomb Repressive Complex 1 and 2 (PRC1 and PRC2) (Margueron & Reinberg, 2011). PRC2 catalyzes the trimethylation of histone H3 lysine 27 (H3K27me3), a key repressive epigenetic mark in higher organisms (Cao et al., 2002; Hansen et al., 2008; Lafos et al., 2011); whereas PRC1 catalyzes the monoubiquitination of histone H2A lysine 119 (H2AK119ub) (Cao & Zhang, 2004).

The subunits of PRC2 are highly conserved in multicellular organisms, but the number of genes that encode each subunit varies among species (Mozgova & Hennig, 2015). The *Drosophila* PRC2 has four main components: Enhancer of zeste (E(z)), Suppressor of zeste 12 (Su(z)12), Extra sex comb (Esc), and p55 (Margueron & Reinberg, 2011; Schwartz & Pirrotta, 2007). In human, two copies of E(z)—*EZH1* and *EZH2* exist (Ciferri et al., 2012). In *Arabidopsis*, there are three homologous genes for the E(z) subunit, *CURLY LEAF* (*CLF*), *SWINGER* (*SWN*), and *MEDEA* (*MEA*); three for Su(z)12, *EMBRYONIC FLOWER 2* (*EMF2*), *VERNALIZATION 2* (*VRN2*), and *FERTILIZATION INDEPENDENT SEED 2* (*FIS2*); one gene for Esc, *FERTILIZATION INDEPENDENT ENDOSPERM* (*FIE*); and five genes for p55, *MULTICOPY SUPRESSOR OF IRA 1‐5* (*MSI1‐5*) (Whitcomb, Basu, Allis, & Bernstein, 2007).

In *Arabidopsis*, PRC2 proteins have been shown to play vital roles in many growth and developmental processes (Bouyer et al., 2011; Jiang, Wang, Wang, & He, 2008; Li et al., 2015; Muller‐Xing, Clarenz, Pokorny, Goodrich, & Schubert, 2014; Tang, Lim, et al., 2012). For instance, *CLF* has long been known to be necessary for the control of leaf and flower morphology, likely through repressing *AGAMOUS* (*AG*) and *SHOOT MERISTEMLESS* (*STM*) (Goodrich et al., 1997; Schubert et al., 2006). *CLF* is also necessary for *WUSCHEL* (*WUS*) repression, contributing to the termination of floral stem cells (Liu et al., 2011), and is required for maintaining root meristem activity by antagonizing the chromatin remodeler PICKLE (PKL) (Aichinger, Villar, Di Mambro, Sabatini, & Köhler, 2011). Unlike the *clf* mutants that display severe phenotypes including dwarfism, curly leaf, and early flowering, the *swn* mutants only show weak changes during vegetative phase transition (Xu, Hu, Smith, & Poethig, 2016; Xu, Guo, et al., 2016). However, the *clf swn* double mutants lose the capacity to differentiate and form massive somatic embryo‐like structures (Chanvivattana et al., 2004; Farrona et al., 2011; Lu, Cui, Zhang, Jenuwein, & Cao, 2011). These prior works have clearly demonstrated the importance of CLF and SWN in plant development, but mostly based on mutant examination and single gene analyses. Meanwhile, although a number of H3K27me3 genome‐wide profiling have been reported (Carter et al., 2018; Cui et al., 2016; Li et al., 2015, 2016; Luo et al., 2013; Wang et al., 2016; Yang et al., 2016), works on genome‐wide occupancy of the CLF and SWN proteins are rather limited. The recently published CLF ChIP‐chip data were based on a transgenic line in which CLF was driven by a constitutive promoter (Xiao et al., 2017). Therefore, there is a pressing need to profile their genome‐wide occupancy for the plant biology community to dissect their functions comprehensively.

Here, we generated *Arabidopsis* transgenic lines expressing tagged CLF or SWN under their native promoters, respectively; and performed ChIP‐seq analyses. We also profiled the genome‐wide changes of H3K27me3 and transcriptomes in the *clf*,* swn*, and *clf swn* mutants. These genomics data would greatly facilitate the deciphering of the detailed roles of plant PcG proteins in various developmental processes.

## 2 EXPERIMENTAL PROCEDURES

### 2.1 Plant materials and growth conditions

WT and the mutants, *clf‐29* (SALK_021003) and *swn‐4* (SALK_109121) of *Arabidopsis* used in this study were all in the Columbia (Col‐0) background, and have been described previously (Bouveret, Schönrock, Gruissem, & Hennig, 2006; Wang, Tyson, Jackson, & Yadegari, 2006). For all experiments, the seeds were stratified for 3 days at 4°C in darkness before sown on soil or on agar plates containing 4.3 g/L Murashige and Skoog (MS) nutrient mix (Sigma‐Aldrich), 1.5% sucrose (pH 5.75), and 0.8% agar. Plants were grown in the long‐day growth room (16‐hr light/8‐hr dark) at 22°C. Transfer DNA (T‐DNA) insertion mutants were obtained from the *Arabidopsis* Biological Resource Center (ABRC). The homozygous T‐DNA insertion mutants were identified by PCR‐based genotyping. The 2‐week‐old (counted when the seeds were transferred into the growth room after stratification) plants grown on synthetic media under 16‐hr light/8‐hr dark condition were used for all analyses unless otherwise specified. All primers are listed in Supporting Information Table S4.

### 2.2 Plasmid constructs and transgenic plants

Two bacterial artificial chromosome (BAC) clones named F26B6 and T10M13 that harbor the *CLF* and *SWN* locus, respectively, were obtained from ABRC. The BAC plasmids were isolated using QIAGEN Large‐Construct Kit, and then used as the templates to amplify the *CLF* and *SWN* genomic sequence including the promoter/regulatory region. The PCR products were purified using the GenepHlow Gel/PCR Kit, and were then subcloned into the binary vector *pMDC107* between the *Pme*I and *Asc*I sites to obtain *pMDC107‐gCLF* and *pMDC107‐gSWN*. The new constructs were sequenced to confirm that the *CLF* (or *SWN*) and *GFP* coding sequences were in‐frame. The constructs were introduced into *Agrobacterium tumefaciens* strain *GV3101*, which were then used to transform into the *clf‐29* and *swn‐4* plants using the floral dip method (Clough & Bent, 1998), respectively. Homozygous transgenic lines with each homozygous genetic background were selected from T3 generation, in which the functional GFP‐tagged proteins were detected.

### 2.3 Flowering time measurement

WT, mutants, and transgenic plants were grown side by side in soil under the long‐day condition (16‐hr light/8‐hr dark) at 22°C. The number of rosette leaves was counted when the inflorescence stem grew to 1 cm in length. At least 10 plants for each genotype were analyzed and the experiment was repeated three times independently.

### 2.4 Western blot

Two grams of 2‐week‐old seedlings were collected, and nuclei were isolated according to the ChIP protocol (Gendrel, Lippman, Martienssen, & Colot, 2005) but without the tissue fixation step. The nuclear protein was released by dissolving the nuclei preparation in 300 μl of lysis buffer (50 mM Tris‐HCl, 10 mM EDTA, 1% SDS, and 1× protease inhibitors) and then sonicated. The protein solution was centrifuged at 16,000 g for 10 min at 4°C to remove debris. Proteins were resolved on a 4%–20% Mini‐PROTEAN TGX Precast Protein Gel (Bio‐Rad) by electrophoresis and detected by antibody to GFP (Abcam, ab290; 1:20,000 dilution) and histone H4 (Millipore, 07‐108; 1:20,000 dilution). Histone H4 was used as the loading control.

### 2.5 Chromatin immunoprecipitation (ChIP) assay

ChIP experiments were carried out as described (Chen et al., 2017; Gendrel et al., 2005; Li et al., 2016) with minor modifications. For the ChIP with the transgenic plants *clf‐29 pCLF::CLF‐GFP* and *swn‐4 pSWN::SWN‐GFP*, another transgenic line that carries GFP only in WT genetic background (*p35S::GFP*) was used as the negative control. Typically, 5 g of plant materials (2‐week‐old seedlings or 2‐week‐old *clf swn* double mutants) grown on agar plates were harvested, which were then cross‐linked with 1% formaldehyde for 20 min under vacuum and ground into fine powder in liquid nitrogen. The chromatin was isolated and sheared into 200–800 bp fragments by sonication. The sonicated chromatin was incubated with anti‐GFP (ab290, Abcam) or anti‐H3K27me3 (07–449, Millipore) antibodies overnight at 4°C with gentle rotating. Then the MinElute PCR Purification Kit (Cat# 28004, Qiagen) was used to recover the precipitated DNA according to the manufacturer's instruction. The ChIP DNA was used for Illumina single‐end (1 × 50 bp) sequencing or qPCR. ChIP‐qPCR was performed with three biological replicates, and results were generated as percentage of input ‐DNA according to the Champion ChIP‐qPCR user manual (SABioscience). DNA quantity and quality were checked using a Qubit fluorometer (ThermoFisher Scientific).

### 2.6 ChIP‐seq and data analyses

At least 2 ng of each ChIP DNA was used to construct ChIP‐seq library, and two biological replicates for each sample. End repair, adapter ligation and amplification were performed using the Illumina Genomic DNA Sample Prep kit according to the manufacturer's protocol. The HiSeq 2500 platform was used for high‐throughput sequencing of the libraries. Sequence data were analyzed essentially as previously described (Chen et al., 2017; Li et al., 2015, 2016). Briefly, after sequencing, the raw sequence reads were cleaned by removing bases with low quality score and cutting sequencing adapter followed by filtering out short reads. Then the cleaned sequence reads were mapped to the *Arabidopsis* genome (TAIR10) (Lamesch et al., 2012) by Bowtie mapper (Langmead, Trapnell, Pop, & Salzberg, 2009) with default mismatch parameters and retaining only reads that can be mapped uniquely to the genome for further analyses. The number of ChIP‐seq reads for all experiments in this study is listed in Supporting Information Table S5. To identify reads enriched regions (peaks), MACS 1.4 (Zhang et al., 2008) was employed to perform peak calling with default settings. High‐confidence target regions were defined as strict overlap of the MACS peaks from two biological replicates. Then the data were imported to Integrated Genome Browser (IGB) (Nicol, Helt, Blanchard, Raja, & Loraine, 2009) for visualization. Peak analyses were performed using PeakAnalyzer (Salmon‐Divon, Dvinge, Tammoja, & Bertone, 2010). The PeakAnnotator was employed to identify functional elements proximal to peak loci though the Nearest Downstream Gene (NDG) subroutine. The target gene of each peak was defined as the genes closest to a given peak localized around the gene body (from 1 kb upstream of TSSs to TTSs). The gene annotation file was downloaded from the EnsemblPlants homepage ( https://plants.ensembl.org/index.html). The SICER program (window size = 600, gap size = 200, false discovery rate (FDR) ≤ 0.01) (Zang et al., 2009) was used to quantitatively compare the H3K27me3 levels of WT and mutants. Peaks with at least a two‐fold change were kept for further analyses. To identify DNA motifs enriched at CLF‐ and SWN‐bound sites, 300 bp sequence surrounding each peak summit (150 bp upstream and downstream, respectively) was extracted and searched for enriched DNA motifs using the DREME/MEME software suite ( http://meme-suite.org/tools/meme-chip) (Machanick & Bailey, 2011) with default settings. The distribution of MACS peaks was performed by ChIPseek, a web‐based analysis tool ( http://chipseek.cgu.edu.tw) (Chen et al., 2014), with default settings. Briefly, the information of chromosome, start site, and end site for each peak was fed to ChIPseek, based on the annotation file from UCSC tair10 assembly, the location of each peak was further grouped into these categories: promoter‐TSS (1 kb upstream of TSS), intergenic, exon, intron, 5′ UTR, 3′ UTR, and TTS. Heat maps of ChIP‐seq were generated using the computeMatrix and plotHeatmap utilities in deepTools2 (Ramirez et al., 2016). The functional annotation was performed using the online “AgriGo” Gene Ontology (GO) analysis toolkit ( http://bioinfo.cau.edu.cn/agriGO/) with default settings.

### 2.7 RNA extraction

Total RNA was isolated from ~50 mg of 2‐week‐old plants (WT and mutants) using the RNeasy Plant Mini kit (Qiagen) according to supplier's instruction. Total RNA was treated with RNase‐free DNaseI kit (Life Techologies).

### 2.8 RNA‐seq

A total amount of 1.5 μg RNA per sample was used as input material for the RNA sample preparations. Sequencing libraries were generated using NEBNext^®^ UltraTM RNA Library Prep Kit for Illumina^®^ (NEB, USA) following manufacturer's recommendations and index codes were added to attribute sequences to each sample. The libraries were sequenced on an Illumina Hiseq 4000 platform and 150 bp paired‐end reads were generated. Three independent biological replicates of each sample were sequenced separately. The raw sequence reads were cleaned by removing bases with low quality score and cutting sequencing adapter followed by filtering out short reads (Supporting Information Table S6). The cleaned reads were mapped to the TAIR10 *Arabidopsis* genome using TopHat v2.0.4 with default settings, except that a minimum intron length of 20 bp and a maximum intron length of 4,000 bp were used. Transcript assembly and calculations to identify differentially expressed genes were carried out by the Cufflinks package (Trapnell et al., 2012). Briefly, the alignment files after running TopHat were provided to Cufflinks to generate a transcriptome assembly for each sample. Then these assemblies were merged together using the Cuffmerge utility. The merged assembly provided a uniform basis for calculating transcript expression in each sample. The reads and the merged assembly were fed to Cuffdiff, where expression levels were calculated and the statistical significance of changes was tested. Genes with at least 1.5‐fold change in expression (FDR = 5%, *p* value < 0.05) were considered to be expressed differentially among these samples in this study.

## 3 RESULTS

### 3.1 Genome‐wide occupancy of CLF and SWN

**Figure 1:**
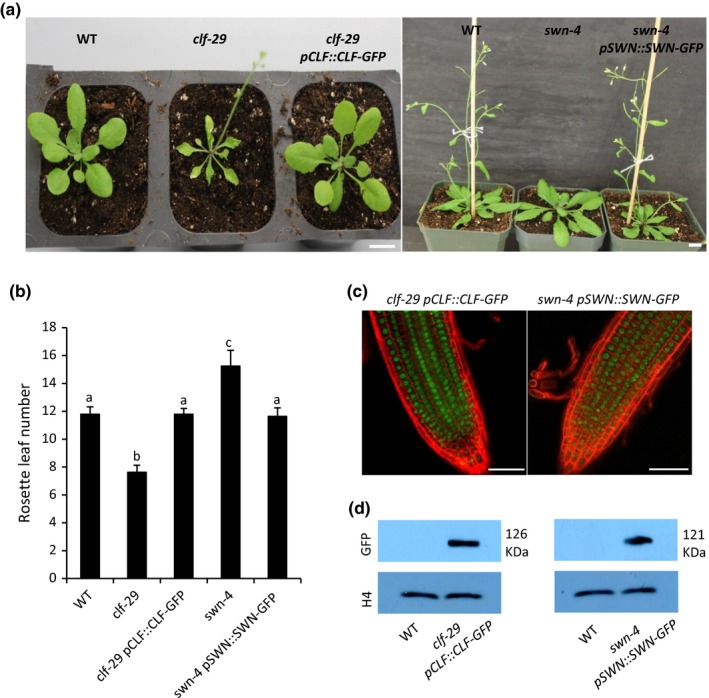
The transgenic lines expressing GFP‐tagged CLF or SWN. (a) Plant photos showing complementation of the *clf‐29* and *swn‐4* flowering phenotypes by the CLF‐ and SWN‐GFP fusion genes driven by their native promoters (*pCLF::CLF‐GFP* and *pSWN::SWN‐GFP*), respectively. Scale bar: 1.0 cm. (b) Rosette leaf number at bolting of plants in different genetic backgrounds at 22°C under long‐day condition. Error bars indicate standard deviation from at least 30 plants. Lowercase letters indicate significant differences between genetic backgrounds, as determined by Post‐hoc Tukey's HSD test. (c) GFP signals detected by confocal microscopy in 4‐day‐old *clf‐29 pCLF::CLF‐GFP* and *swn‐4 pSWN::SWN‐GFP* roots. Scale bar: 50.0 μm. (d) Western blot analysis of nuclear extracts from 2‐week‐old *clf‐29 pCLF::CLF‐GFP* and *swn‐4 pSWN::SWN‐GFP* seedlings. Antibodies used: GFP (anti‐GFP; top) and H4 (anti‐histone H4; bottom). WT: wild type.

**Figure 2:**
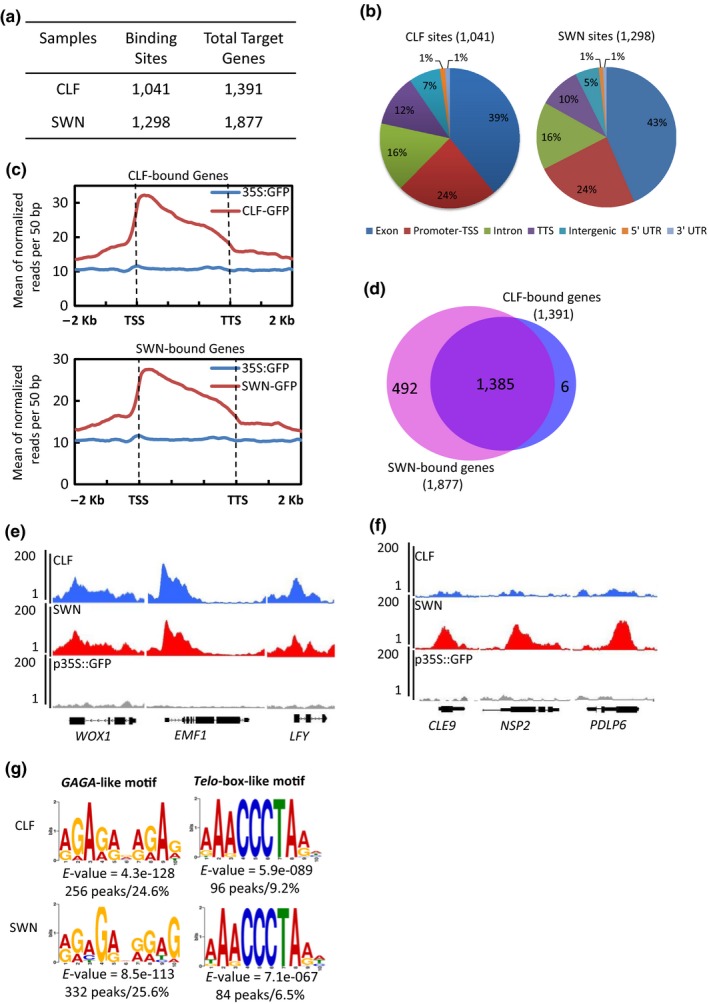
Genome‐wide occupancy of CLF and SWN. (a) Table showing the numbers of CLF and SWN binding sites and target genes. (b) Pie charts showing the distribution of CLF and SWN at annotated genic and intergenic regions in the genome. (c) Mean density of CLF/SWN occupancy at all target genes. Plotting regions were scaled to the same length as follows: 5′ ends (−2 kb to transcription starting site (TSS)) and 3′ ends (transcription stop site (TTS) to downstream 2 kb) were not scaled, and the gene body was scaled to 3 kb. (d) Venn diagram showing the overlap between the genes occupied by CLF and those by SWN. (e, f) ChIP‐seq genome browser views of CLF and SWN co‐occupancy (e) and SWN unique occupancy at selected genes (f). Gene structures are shown underneath each panel. (g) Two motifs enriched in CLF and SWN peaks. The number and percent of peaks containing the motifs are shown.

To profile the genome‐wide targets of CLF, we generated a transgenic line expressing GFP‐tagged CLF (CLF‐GFP) under the control of its own promoter in the *clf‐29* genetic background (*clf‐29 pCLF::CLF‐GFP*). The transgene could fully rescue the phenotypes of the *clf ‐29* mutant (Figure 1a,b). The GFP signal could be observed by confocal microscopy (Figure 1c), and the CLF‐GFP protein could be detected by Western blot (Figure 1d). Then ChIP‐seq was performed with 2‐week‐old transgenic plants. A transgenic line expressing *GFP* only under the tobacco mosaic virus 35S promoter (*p35S::GFP*) was used as the negative control. Two independent biological replicates of ChIP DNAs were sequenced. We observed a good correlation between the two replicates (the Pearson coefficient is 0.89) (Supporting Information Figure S1). A total of 1,041 genomic regions (peaks), corresponding to 1,391 genes, were bound by CLF (Figure 2a; Supporting Information Data S1 and S2). More than half of the CLF binding sites were located in promoter regions and exons, and about 16% of the peaks were in introns (Figure 2b). In general, the CLF binding signals were found to be the strongest at transcription start sites (TSSs), and gradually declined toward transcription termination sites (TTSs) (Figure 2c).

Similarly, we also generated a SWN‐GFP transgenic line in the *swn‐4* background (*swn‐4 pSWN::SWN‐GFP*) and the transgene could fully rescue the late flowering phenotype of *swn‐4* (Figure 1a,b). We could observe the GFP signal by confocal microscopy (Figure 1c). The SWN‐GFP protein could be detected by Western blot (Figure 1d). Two independent biological ChIP‐seq experiments were carried out using 2‐week‐old *swn‐4 pSWN::SWN‐GFP* seedlings. The two replicates were well correlated with a high Pearson coefficient (0.92) (Supporting Information Figure S1). We found that SWN binds to 1,298 genomic sites, corresponding to 1,877 genes (Figure 2a; Supporting Information Data S1 and S2). About 43% of the SWN binding sites were found in exons, followed by 24% in promoter regions and 16% in introns (Figure 2b). The SWN ChIP‐seq signals were generally enriched at TSSs, and gradually decreased toward TTSs (Figure 2c).

Then we compared the binding profiles of CLF and SWN. The two H3K27 methyltransferases exhibited highly similar chromatin‐associated profiles (Figure 2c; Supporting Information Table S1), which are consistent with previously observed genetic redundancy between the two genes (Chanvivattana et al., 2004; Farrona et al., 2011). Furthermore, we analyzed the distribution of CLF and SWN binding along each of the chromosomes. As shown in Supporting Information Figure S2, CLF and SWN predominantly bind to euchromatic regions. Almost all of the CLF‐bound genes were also bound by SWN (1,385 out of the 1,391 CLF targets); only 6 genes were bound by CLF uniquely, while nearly 500 more genes bound by SWN solely (Figure 2d; Supporting Information Data S2). ChIP signals at some selected loci representing the CLF and SWN co‐targets and SWN unique targets were shown in Figure 2e,f, respectively. The ChIP‐seq results were validated by ChIP‐quantitative PCR (ChIP‐qPCR) analyses on several randomly selected target genes (Supporting Information Figure S3). Furthermore, we also compared our CLF/SWN target genes with FIE target genes by taking advantage of the published datasets (Deng et al., 2013; Xiao et al., 2017). As shown in Supporting Information Figure S4, CLF/SWN and FIE share a large fraction of targets (Supporting Information Data S3).This result is understandable, given the fact that these experiments were carried out with plants of different ages. It might also imply that CLF/SWN and FIE probably have distinct functions, although the *clf swn* double mutants share similar phenotypes with the *fie* mutants (Bouyer et al., 2011; Chanvivattana et al., 2004). In mammals, similarly, a partial overlap between the occupancy of H3K27 methyltransferases EZH1 and EED has been reported recently (Bodega et al., 2017).

### 3.2 Identification of two DNA motifs from CLF and SWN binding sites

To examine whether any particular DNA motifs are enriched in the identified CLF and SWN binding regions, stringent motif searches were performed and two DNA motifs were identified for both CLF and SWN, a *GAGA*‐like motif and a *Telo*‐box‐like motif (Figure 2g; Supporting Information Table S2). The *GAGA*‐like is the most enriched motif (24.6% of CLF peaks; 25.6% of SWN peaks) with a high significance (*E*‐value ≤8.5e−113). In *Drosophila*, there are many PcG response elements (PREs) including GA‐repeats that are recognized by GAGA factors (GAFs) (Farkas et al., 1994; Hodgson, Argiropoulos, & Brock, 2001; Katsani, Hajibagheri, & Verrijzer, 1999). The *Telo*‐box motif is a short sequence identical to plant telomere‐repeat units (AAACCCTA)n, which was originally observed in the 5′ flanking regions of genes encoding ribosomal proteins and the translation elongation factor EF1α (Gaspin, Rami, & Lescure, 2010). Although not highly enriched in the CLF and SWN peaks (9.2% of CLF peaks; 6.5% of SWN peaks), this motif was discovered with a high significance (*E*‐value ≤7.1e−067). The *Telo*‐box motif has been shown very recently to be recognized by the telomere‐repeat factors (TRBs) which then recruit CLF/SWN through direct physical interaction with CLF/SWN (Zhou et al., 2018). Of note, the two motifs were also previously identified in the genomic binding regions of other PcG proteins, FIE and LHP1 (Deng et al., 2013; Molitor et al., 2016; Xiao et al., 2017; Zhou, Hartwig, James, Schneeberger, & Turck, 2016).

### 3.3 Functions of CLF and SWN target genes in plant growth and development

**Figure 3:**
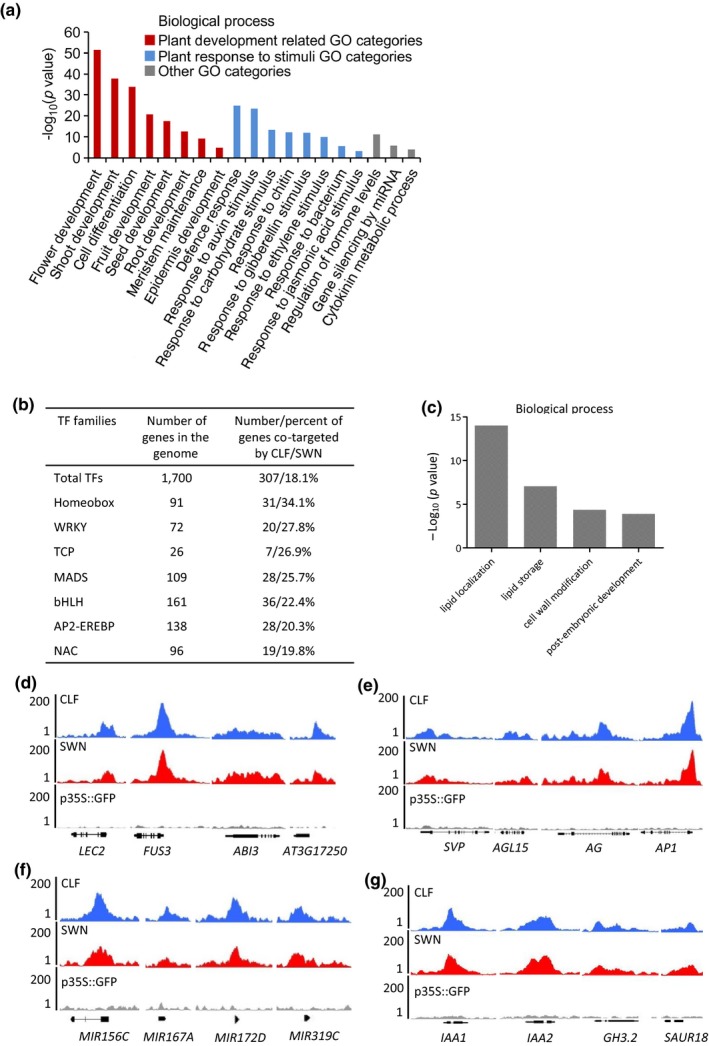
Functional categorization of CLF and SWN target genes. (a) Gene Ontology (GO) analysis of CLF and SWN co‐target genes. (b) Table showing the transcription factor gene families that are co‐targeted by CLF and SWN. The transcription factor gene families can be found at the *Arabidopsis* genome resources website ( http://arabidopsis.med.ohio-state.edu/AtTFDB/). (c) GO analysis of the SWN unique target genes. (d‐g) ChIP‐seq genome browser views of CLF and SWN occupancy at selected loci; *p35S::GFP* transgenic plants as the negative control. Gene structures are shown underneath each panel.

To reveal the potential functions of CLF and SWN target genes, we performed a Gene Ontology (GO) analysis and found that CLF and SWN preferentially bind to genes involved in developmental pathways and abiotic and biotic stress responses (Figure 3a; Supporting Information Data S4). Particularly, genes encoding members of several transcription factor (TF) families that are involved in developmental processes and stress responses are markedly overrepresented in the CLF and SWN co‐targets, such as the Homeobox, WRKY, and MADS families (Figure 3b; Supporting Information Table S3). In contrast, the SWN unique targets are enriched in those involved in lipid localization/storage, cell wall modification, and post‐embryonic development (Figure 3c; Supporting Information Data S4).

Shown in Figure 3d‐g are ChIP‐seq signals at selected gene loci that play important roles in several growth and developmental processes. The major seed regulatory genes including *LEAFY COTYLEDON2* (*LEC2*), *FUSCA3* (*FUS3*), and *ABSCISIC ACID INSENSITIVE3* (*ABI3*) (Tang et al., 2008; Tang, Bian, et al., 2012; Tang, Lim, et al., 2012; To et al., 2006) and seed maturation genes, such as *AT3G17520* which belongs to the late embryogenesis abundant protein (LEA) family (Lin, Pajak, Marsolais, McCourt, & Riggs, 2013) were co‐bound by CLF and SWN (Figure 3d). These findings suggest that CLF and SWN may be involved in the repression of the seed maturation program at seedling stage by directly targeting to the key maturation genes. CLF/SWN binds to several major floral transition genes including *SHORT VEGETATIVE PHASE* (*SVP*) and *AGAMOUS‐LIKE 15* (*AGL15*), which belong to type II MADS box gene family (Chiang, Barua, Kramer, Amasino, & Donohue, 2009; Harding, Tang, Nichols, Fernandez, & Perry, 2003; Hartmann et al., 2000; Li et al., 2015; Michaels et al., 2003; Smaczniak, Immink, Angenent, & Kaufmann, 2012), and flower organ identity genes like *AGAMOUS* (*AG*) and *APETALA1* (*AP1*) (Figure 3e), indicating that CLF and SWN play an important role in silencing the flower development program during vegetative growth. It is well documented that the antagonistic activities of microRNAs *miR156* and *miR172* coordinate the juvenile‐to‐adult leaf transition; *miR319* represses the onset of senescence by targeting the *TEOSINTE BRANCHED1*,* CYCLOIDEA*, and *PCF* (*TCP*) transcription factors; and *miR167* is involved in the regulation of auxin homeostasis and adventitious rooting (Chen, 2004; Rubio‐Somoza & Weigel, 2011; Schommer et al., 2008; Wu et al., 2009). The gene loci encoding these microRNAs are all co‐targets of CLF and SWN (Figure 3f). Plant hormones such as auxin mediate many growth and developmental processes (Bari & Jones, 2009). We noticed that CLF and SWN bind to genes that are involved in hormonal signaling pathways, for example, genes that encode key players in auxin signaling pathways, the *Aux/IAA* transcriptional repressors, *GH3* family, and *SMALL AUXIN UPREGULATED RNA* (*SAUR*) family (Woodward & Bartel, 2005) (Figure 3g).

### 3.4 Genome‐wide profiling of H3K27me3 in *clf‐29*,* swn‐4*, and *clf‐29 swn‐4*

**Figure 4:**
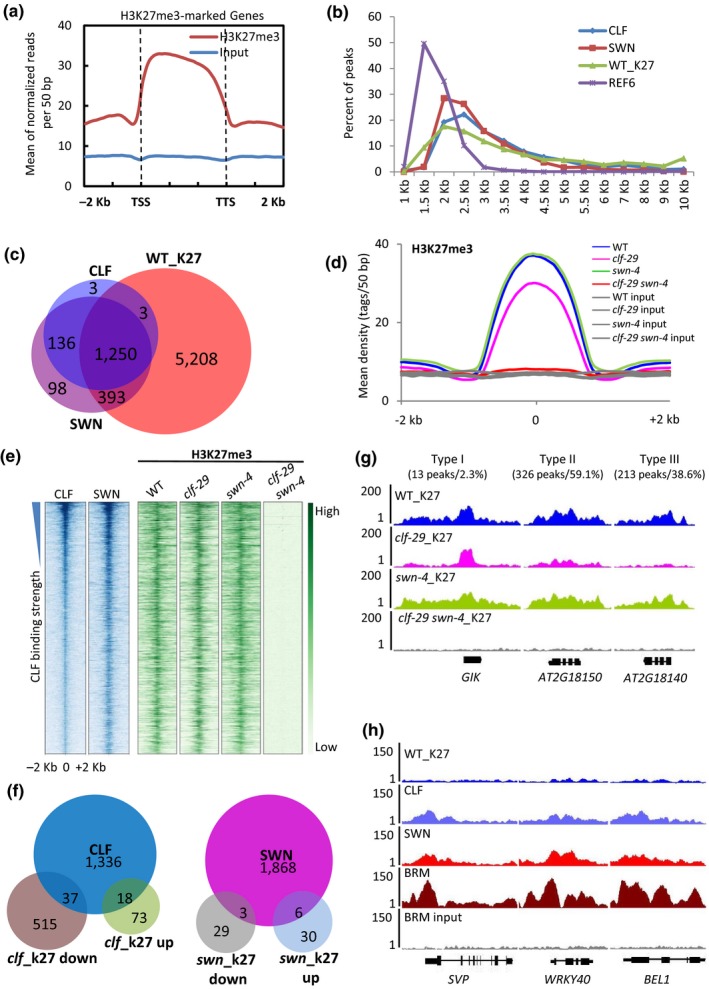
Genome‐wide profiling of H3K27me3 in *clf‐29*,* swn‐4*, and *clf‐29 swn‐4*. (a) Mean density of H3K27me3 occupancy at all target genes in WT. Plotting regions were scaled to the same length as follows: 5′ ends (−2 kb to transcription starting site (TSS)) and 3′ ends (transcription stop site (TTS) to downstream 2 kb) were not scaled, and the gene body was scaled to 3 kb. (b) The width ranges for CLF, SWN, and WT_K27 (WT_H3K27me3) peaks. The *x* axis shows the width of peaks within the ranges (e.g. 1 kb: width <1 kb; 1.5 kb: 1 kb ≤ width ≤ 1.5 kb). The *y* axis represents the percent of peaks in each range. The REF6 peaks were used as representative “narrow peaks” (Li et al., 2016). (c) Venn diagram showing the overlap among the genes marked with H3K27me3 in WT (WT_K27) and those occupied by CLF or SWN. (d) Mean density of H3K27me3 levels in WT,* clf‐29*,* swn‐4*, and *clf‐29 swn‐4*. Inputs from all backgrounds are shown in gray. The average signal within 2 kb genomic regions flanking the center of the H3K27me3 peaks in WT is shown. (e) Heat maps representing the co‐occupancy of CLF and SWN in the genome (blue, left), and the H3K27me3 levels in WT,* clf‐29*,* swn‐4*, and *clf‐29 swn‐4* (green, right). Each horizontal line represents a CLF/SWN binding peak or H3K27me3 peak. Columns show the genomic region surrounding each peak summit. Signal intensities are indicated by the shade of blue or green. (f) Venn diagrams showing the overlaps between the genes occupied by CLF and the genes with decreased (*clf*_K27 down) and increased H3K27me3 levels (*clf*_K27 up) in *clf‐29* (left); and between the genes occupied by SWN and the genes with decreased (*swn*_K27 down) and increased H3K27me3 levels (*swn*_K27 up) in *swn‐4* (right). (g) ChIP‐seq signals at representative genomic loci showing three distinct types of H3K27me3 reduction pattern in *clf‐29* compared to WT. Gene structures are shown underneath the panel. (h) ChIP‐seq genome browser views of selected genes showing the distribution of H3K27me3 in WT (WT_K27), and the occupancy of CLF, SWN, and BRM (Li et al., 2016). BRM ChIP‐seq input signals at these genes are also shown as the negative control. Gene structures are shown underneath the panel. WT: wild type.

To further examine the functional redundancy between CLF and SWN in *Arabidopsis* seedlings, the genome‐wide distributions of H3K27me3 in WT, *clf‐29*,* swn‐4*, and *clf‐29 swn‐4* were examined. In WT, we were able to identify 6,854 genes marked by H3K27me3 (Supporting Information Data S5 and S6), which largely overlap with previously published genome‐wide profiles (Supporting Information Figure S5a) (Li et al., 2015; Wang et al., 2016). By analysing the distribution pattern of H3K27me3 peaks over annotated genomic features, we found that the marker has an even distribution from TSSs to TTSs (Figure 4a). Nearly 63% of the peaks were localized in genic regions (Supporting Information Figure S5b). The peaks of H3K27me3 and CLF/SWN are very similar in terms of their width range and are generally much broader than those of REF6, a DNA binding factor that shows typical narrow binding peaks (Figure 4b) (Li et al., 2016).

When comparing occupancy profile of H3K27me3 with that of CLF/SWN, it is obvious that the vast majority of the CLF and SWN binding genes were marked by H3K27me3 (1,253 out of 1,391 for CLF; 1,643 out of 1,877 for SWN) (Figure 4c; Supporting Information Data S6), consistent with the notion that both enzymes directly bind to their target loci to deposit H3K27me3. To our surprise, three quarters of the H3K27me3‐marked genes were not occupied by either CLF or SWN in WT seedlings (Figure 4c). The global level of H3K27me3 in *clf‐29* was significantly lower than that in WT, while in *swn‐4*, it was almost identical to that in WT (Figure 4d). In the *clf‐29 swn‐4* double mutant, nearly no H3K27me3 could be detected (Figure 4d,e), which is consistent with previous reports (Lafos et al., 2011; Zhou, Romero‐Campero, Gómez‐Zambrano, Turck, & Calonje, 2017). To eliminate the possibility that the loss of H3K27me3 was due to the change of nucleosome distribution, we checked the H3 distribution in the *clf‐29 swn‐4* double mutant. The H3 distribution levels in *clf‐29 swn‐4* were very similar to those in WT, with slight reduction at some loci examined (Supporting Information Figure S6). Taken together, these results suggest that CLF and SWN are probably the only enzymes that catalyze H3K27me3 at this developmental stage.

Comparing the H3K27me3 levels in *clf‐29* and WT, we identified 552 genes at which the levels of H3K27me3 were reduced at least two‐fold in *clf‐29*, and 91 genes that showed at least two‐fold increase (Supporting Information Data S7). However, only 37 out of the 552 genes were CLF direct targets (Figure 4f). In *swn‐4*, the changes of H3K27me3 level were only detected at 68 genes (32 increase and 36 decrease; two‐fold cut off) (Figure 4f; Supporting Information Data S7), which were consistent with the findings shown in Figure 4d that the global level of H3K27me3 in *swn‐4* seemed to be unchanged relative to that of WT. After close examination of the regions with reduced H3K27me3 in *clf‐29*, we identified three types of reduction pattern (Figure 4g). In type I, loss of *CLF* had only weak or no effect on the H3K27me3 peak summit, but caused drastic reduction of H3K27me3 in flanking regions (type I, Figure 4g). The remaining H3K27me3 signals at the summit completely disappeared in the *clf‐29 swn‐4* double mutant, as shown in the ChIP‐seq described as above and confirmed by ChIP‐qPCR (Supporting Information Figure S7). In type II, a partial reduction of H3K27me3 across the entire H3K27me3‐marked region was observed in *clf‐29*, and the remaining signal was completely abolished in the *clf‐29 swn‐4* double mutant (Supporting Information Figure S7). In type III, we saw almost complete loss of H3K27me3 in *clf‐29* (Supporting Information Figure S7), suggesting a non‐redundant role of CLF in catalyzing H3K27me3 at these genes.

Noticeably, there is a small group of genes that are CLF/SWN targets, but showed no or very low levels of H3K27me3 (Figure 4h). This is reminiscent of our previous findings at some genomic loci such as the floral repressor locus *SVP* (Li et al., 2015). In that work we demonstrated that the chromatin remodeler BRM could promote gene expression by limiting the deposition of H3K27me3 through suppressing the recruitment and/or the activity of CLF/SWN. Therefore, to find out whether the lack of H3K27me3 deposition is due to the presence of BRM, we checked the BRM occupancy at those CLF/SWN targets (Archacki et al., 2017; Li et al., 2016). Indeed, we saw nearly half of these genes (63 out of 136) were bound by BRM as well (Supporting Information Data S8). ChIP‐seq signals showing the H3K27me3 levels and the occupancy of CLF, SWN, and BRM at several representative loci are presented in Figure 4h.

### 3.5 Transcriptome profiling in *clf‐29, swn‐4,* and *clf‐29 swn‐4*

**Figure 5:**
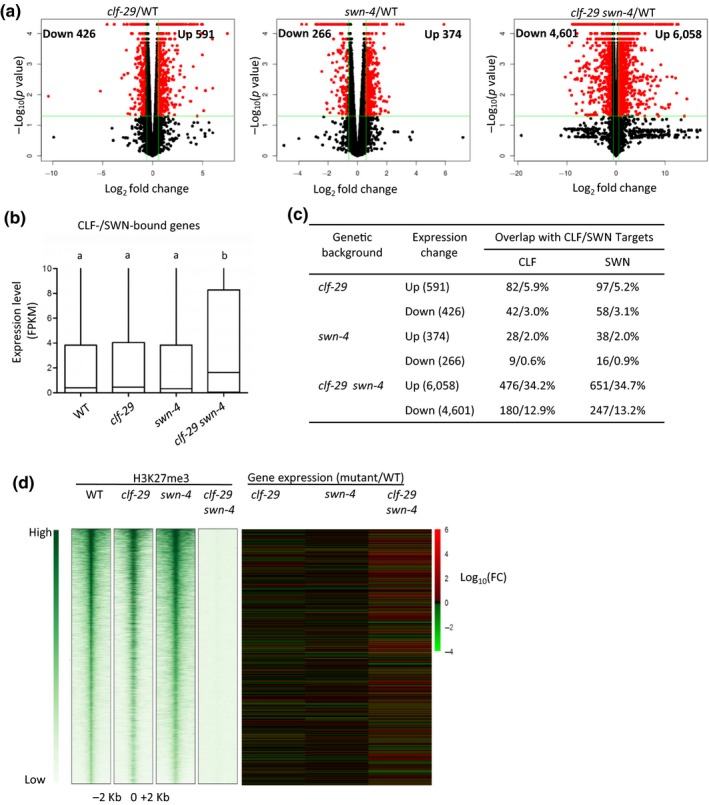
Transcriptome profiling of *clf‐29*,* swn‐4*, and *clf‐29 swn‐4*. (a) Volcano plots displaying significantly up‐regulated and down‐regulated genes in *clf‐29*,* swn‐4*, and *clf‐29 swn‐4* compared to WT (red dots, *p *<* *0.05, fold change > 1.5), respectively. The *x* axis represents the Log2 value of fragments per kilobase per million (FPKM) mapped reads in each mutant/WT, and the *y* axis is the −Log10 of the *p* value for the significance of differential expression. (b) Box plots representing the average expression level (FPKM) of CLF and SWN co‐targets in *clf‐29*,* swn‐4*, and *clf‐29 swn‐4*. Lowercase letters indicate significant differences between genetic backgrounds, one‐way ANOVA. (c) Table showing the percent of overlaps for genes bound by CLF/SWN with differential expression in *clf‐29*,* swn‐4*, and *clf‐29 swn‐4* compared to these in WT. (d) Heat maps illustrating the ChIP‐seq density in WT and mutants (*clf‐29*,* swn‐4*, and *clf‐29 swn‐4*), ranked by H3K27me3 read intensity within ±2 kb of peak summits in WT (green, left), and the RNA‐seq intensity for the H3K27me3‐marked loci in WT with the same order. Each horizontal line represents an H3K27me3 peak. Columns show the genomic region surrounding each peak summit. Signal intensities are indicated by the shade of green. The expression intensity is measured by Log10 (FC), FC = fold change (mutant vs. WT FPKM). WT: wild type.

To further understand the action of CLF and SWN, we performed RNA‐seq experiments to profile transcriptome changes in the *clf‐29*,* swn‐4*, and *clf‐29 swn‐4* mutants and compared to those in WT. In *clf‐29*, we found that 591 genes were transcriptionally up‐regulated compared to WT, whereas the expression of 426 genes was down‐regulated (Figure 5a; Supporting Information Data S9), by at least 1.5‐fold (absolute fold change [FC] ≥ 1.5) at a false discovery rate (FDR) <0.05. In *swn‐4*, a total of 374 genes showed increased expression and 266 genes showed decreased expression compared to WT (Figure 5a; Supporting Information Data S9). Overall, fewer genes showing altered expression were detected in *swn‐4* compared to those in *clf‐29*, which may explain why the *swn‐4* mutants display less severe morphological defects compared to the *clf‐29* mutants. In *clf‐29 swn‐4*, more than 10,000 genes (6,058 up‐regulated; 4,601 down‐regulated) showed differential expression compared to WT (Figure 5a; Supporting Information Data S9). In the WT plants, it is clear that the global expression levels of all the H3K27me3‐marked genes and CLF‐/SWN‐bound genes were lower than the rest of genes in the genome (Supporting Information Figure S8). This observation is expected given the recognized role of PRC2 in repressing gene expression (Wang et al., 2016).

Next, we compared the binding profile of CLF/SWN with the transcriptome data. Generally, the expression of those genes bound by CLF/SWN is similar between the *clf‐29* and *swn‐4* single mutants and WT, but showing significant up‐regulation in the *clf‐29 swn‐4* double mutant (Figure 5b), indicating that *CLF* and *SWN* play partially redundant roles at these loci. Furthermore, nearly one‐third of the CLF/SWN target genes showed up‐regulation in *clf‐29 swn‐4* (Figure 5c). To examine the biological functions of CLF/SWN targets that were up‐regulated in the double mutant, GO analysis was performed. The up‐regulated genes were functionally enriched in biosynthetic process, developmental process, and response to stimulus (Supporting Information Figure S9; Data S10). We also examined whether there is a correlation between the loss of H3K27me3 and the expression levels of transcripts in the *clf swn* double mutants. As shown in Figure 5d, we observed up‐regulation of many genes in *clf‐29 swn‐4*, particularly those that were heavily marked by H3K27me3 in WT plants, but overall there was no clear correlation between the loss of H3K27me3 and the expression levels in *clf‐29 swn‐4*.

## 4 DISCUSSION

Epigenetic regulation of gene expression through histone modifications is fundamental for plant growth and development. In particular, the repressive histone modification H3K27me3 marks a large fraction of the *Arabidopsis* genome dynamically and plays critical roles in plant development (Derkacheva & Hennig, 2014; Gan, Xu, & Ito, 2015; Li et al., 2015; Wiles & Selker, 2017; Zhang et al., 2007). However, the global binding profiles of the two major H3K27me3 methyltransferases, CLF and SWN, have not been determined and compared. Thus, our CLF and SWN ChIP‐seq data, together with the H3K27me3 ChIP‐seq and the transcriptome data will be valuable resources for the plant epigenetics research community. Further, the *CLF‐* and *SWN‐GFP* fusion transgenic lines (driven by their corresponding native promoters) could be used to study the function of PcG proteins in many other developmental processes and stress response pathways in *Arabidopsis*.

In the following, we would like to highlight and discuss several interesting findings from this study. These findings provide new biological insights into the mechanisms underlying PRC2 activities, particularly the functional interplays between CLF and SWN. Our results also shed fresh lights on the roles of PRC2 in two developmental transitions, i.e., flowering and the repression of seed genes during vegetative growth. Further, future investigations are warranted to address many of the new questions arising from this study, which would undoubtedly enhance our understanding of PcG activities and their regulation during plant growth and development.

Our data indicate that CLF and SWN are highly redundant in depositing H3K27me3, and probably they are the only two proteins that are able to catalyze H3K27me3 at the seedling stage. Strikingly, the majority of H3K27me3‐marked regions were not bound by either CLF or SWN. Since CLF and SWN are the enzymes required to catalyze H3K27me3 during seedling stage, therefore, H3K27me3‐marked regions are expected to be occupied by CLF and/or SWN. This apparent discrepancy/mystery could possibly be explained by technical and/or biological reasons. Technically, it would be very hard to detect binding signals by ChIP‐seq if CLF and SWN bind to some regions very weakly. Similar findings were reported in *Drosophila*: genome‐wide comparison of PRC2 and H3K27me3 profiles identified very “weak” PcG binding sites (as the authors called), where H3K27me3 but not PRC2 was detected (Schwartz et al., 2006). Biologically, explanations for the smaller number of CLF and SWN peaks compared to the H3K27me3 peaks could be that CLF and SWN bind to chromatins transiently, they might leave target sites as long as the H3K27me3 is deposited. Supporting this possibility, a fluorescence recovery after photobleaching study (FRAP) in *Drosophila* found that PcG members are exchangeable at specific loci in the genome (Ficz, Heintzmann, & Arndt‐Jovin, 2005). We also need to bear in mind that ChIP‐seq profiling is just a snapshot of the occupancy, but the H3K27me3 marks observed are the culmination of PRC2 activity at all earlier stages, as events that cannot be fully recorded at just one time point.

Another potentially significant finding is about the differential roles of CLF and SWN in depositing the H3K27me3 mark at the same loci. As shown in Figure 4g, we saw genomic sites where CLF and SWN play partially redundant or non‐redundant roles in depositing H3K27me3. Perhaps more interestingly, we also found genomic sites where loss of *CLF* led to a marked reduction of H3K27me3 in flanking regions, but not the peak summit, suggesting that CLF might help the spreading of H3K27me3 to flanking regions from a “core” area. Similar H3K27me3 pattern changes were also reported in previous studies (Li & Cui, 2016; Wang et al., 2016; Yang et al., 2017; Yuan et al., 2016). The remaining H3K27me3 signal at the summit in the *clf‐29* mutants might be catalyzed by SWN. Indeed, the H3K27me3 signal at the summit completely disappeared in the *clf‐29 swn‐4* double mutant (Figure 4g). Thus, SWN might play redundant roles in catalyzing H3K27me3 only at the peak summit, but not the flanking regions. These observations present us questions that warrant future investigation, such as the locus‐specific recruitment of CLF/SWN and their functional interplay in depositing H3K27me3. In terms of locus‐specific recruitment of PcG proteins, this and other recent studies have identified DNA motifs that are enriched in PcG binding sites (Figure 2g) (Deng et al., 2013; Molitor et al., 2016; Xiao et al., 2017; Zhou et al., 2018). Future studies are needed to decipher their roles in mediating the genomic targeting of PcG proteins. One possible way to examine the role of these DNA motifs in recruiting PcG proteins is by using the CRISPR/Cas9 system to disrupt the motif *in vivo* and then check the enrichment levels of PcG proteins (Li et al., 2018). It is also crucial to identify the DNA sequence‐specific players that mediate the locus‐specific recruitment of PcG proteins (Zhou et al., 2018).

Our transcriptome analyses revealed that the expression of more genes was altered in the *clf swn* double mutant than either the *clf* or *swn* single mutants (Figure 5). This observation was consistent with the genetic redundancy of *CLF* and *SWN* and the recognized functions of PcG proteins in repressing gene expression. Although thousands of genes were clearly up‐regulated in the double mutants, we were surprised to find that the expression of many other genes remained unchanged and thus there was no overall correlation between the loss of H3K27me3 and up‐regulation of gene expression. Similar observation was made in our previous transcriptome analysis in *brm* and *ref6* mutants—many BRM/REF6 targets did not show alteration in gene expression (Li et al., 2015). This may be due to the possibility that many other factors also function at these sites, and the gene expression outcome may not be significantly affected by only one of them. In addition, there was almost equal number of genes that showed down‐regulation in the double mutants, which were not likely caused directly by the loss of H3K27me3, but rather indirectly via some intermediate effectors.

It is well known that PcG proteins play important roles in developmental transitions, but the underlying mechanisms are incompletely defined. Here, by integrating CLF/SWN and H3K27me3 ChIP‐seq and RNA‐seq data (Supporting Information Figure S10), we attempted to show how CLF/SWN may act to regulate two developmental transitions, that is, repression of seed genes during vegetative growth and control of flowering time. The seed development regulatory genes *LEC1, LEC2*,* FUS3*,* ABI3,* and the four *2S* and the three *12S* genes (Lin et al., 2013; Tang et al., 2008; Tang, Bian, et al., 2012; Tang, Lim, et al., 2012; To et al., 2006) were all marked by H3K27me3. The levels of H3K27me3 showed no significant changes in either the *clf‐29* or *swn‐4* single mutants, but all completely abolished in the *clf‐29 swn‐4* double mutant, consistent with the depression of these seed genes in the double mutants (Chanvivattana et al., 2004; Farrona et al., 2011; Lu et al., 2011). Although these seed genes were all clearly marked by H3K27me3, the occupancy of CLF/SWN varied considerably. The *2S* and *12S* genes appeared to be mainly bound by SWN, not by CLF. The *LEC2*,* FUS3*, and *ABI3* genes were bound by both CLF and SWN, but the binding intensities varied drastically, with the strongest binding being found at *FUS3*, greatly reduced at *LEC2* and *ABI3*. The *LEC1* gene was bound by SWN only (Supporting Information Figure S10a). These findings suggest that CLF and SWN play redundant, yet differential, roles in repressing the seed maturation program during the seedling stage.

Our data also provide some clues for the early flowering phenotype of the *clf* mutants. As shown in Supporting Information Figure S10b, several genes that encode positive regulators of floral transition such as *AGLs 17*,* 19*,* 24*, and *71*,* SOC1*, and *FT*, showed up‐regulation in *clf‐29*, but not in *swn‐4*. The two flowering repressor‐encoding genes *FLC* and *SVP* showed differential expression (up‐ and down‐regulated, respectively). The increase of *FLC* expression is consistent with the reduction of H3K27me3; and the decrease of *SVP* could be (at least partially) due to the slight increase of H3K27me3 level at this locus in *clf‐29*. As FLC and SVP may actually work together by forming a repressor complex (Li et al., 2008; Mateos et al., 2015), it is reasonable to speculate that the reduction of *SVP* in *clf‐29* would likely result in less amount of the complex and consequently more *FT*. This is likely the case, as we and others have shown that *SVP* represses flowering in a dosage‐sensitive manner (Hartmann et al., 2000; Li et al., 2015). Of note, we and others have consistently found the up‐regulation of *FT* in the *clf‐29* mutants (Farrona et al., 2011; Jiang et al., 2008; Liu et al., 2018). But, unlike the previous studies, we did not find significant CLF/SWN enrichment at this locus (Supporting Information Figure S10b). This apparent inconsistency could be due to the different plant materials used in these independent studies, that is, the *CLF‐GFP* fusion gene used in our work was driven by the native *CLF* promoter, while it was driven by constitutive promoters in other studies.

## CONFLICT OF INTEREST

The authors declare no conflict of interest.

## AUTHOR'S CONTRIBUTION

J.S., C.C., C.L., S.E.K., and Y.C. conceived and designed the experiments; V.N. and Z.C.Y. performed all the Sanger DNA sequencing; J.S. performed all the rest of the experiments; J.S, C.C., R.K.T., S.B., K.Y., and J.L. analyzed ChIP‐seq and RNA‐seq data; J.S., C.C., C.L., and Y.C. wrote the paper.

## ACCESSION NUMBERS

The gene accession numbers that were used in this study are as follows: *CLF* (AT2G23380) and *SWN* (AT4G02020).

ChIP‐seq and RNA‐seq data have been deposited in the National Center for Biotechnology Information GEO database under accession number GSE108960.
