# Supplementary material for: Genome‐wide occupancy of histone H3K27 methyltransferases CURLY LEAF and SWINGER in Arabidopsis seedlings
Source: Plant Direct. 2019 Jan 31;3(1):e00100. doi: 10.1002/pld3.100 (PMC6508855; doi:10.1002/pld3.100)
Supplement: Supplementary file 1 [file PLD3-3-e00100-s001.pdf]

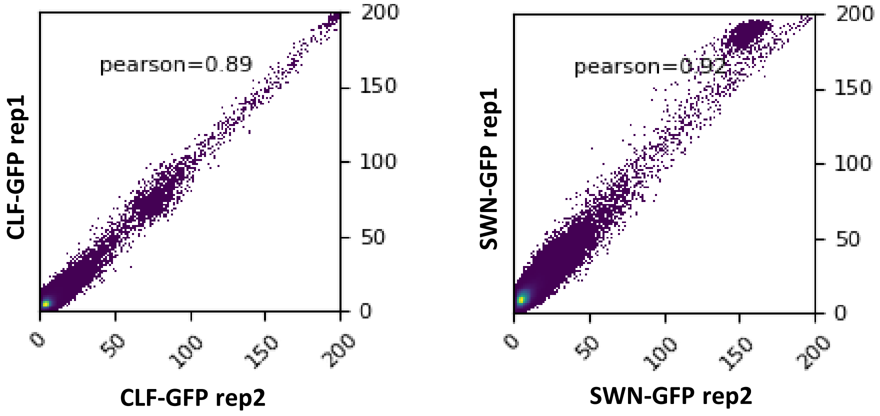

Figure S1. Pearson correlation plots for the two CLF and SWN ChIP-seq replicates. The whole genome is divided into non-overlapped bins (50 bp), and the normalized reads of each replicate (normalization was performed by randomly selecting same number of reads from each replicate) were filled in each bin. The numbers of normalized reads in each bin were plotted. The *x* and *y* axis are the number of reads in each bin for each biological replicate. The correlation between CLF-GFP and SWN-GFP ChIP-seq replicates is very high (median *r* = 0.89 and 0.92, respectively), indicating that both biological replicates can produce reproducible results. The analysis was performed by using the deepTools2 package.

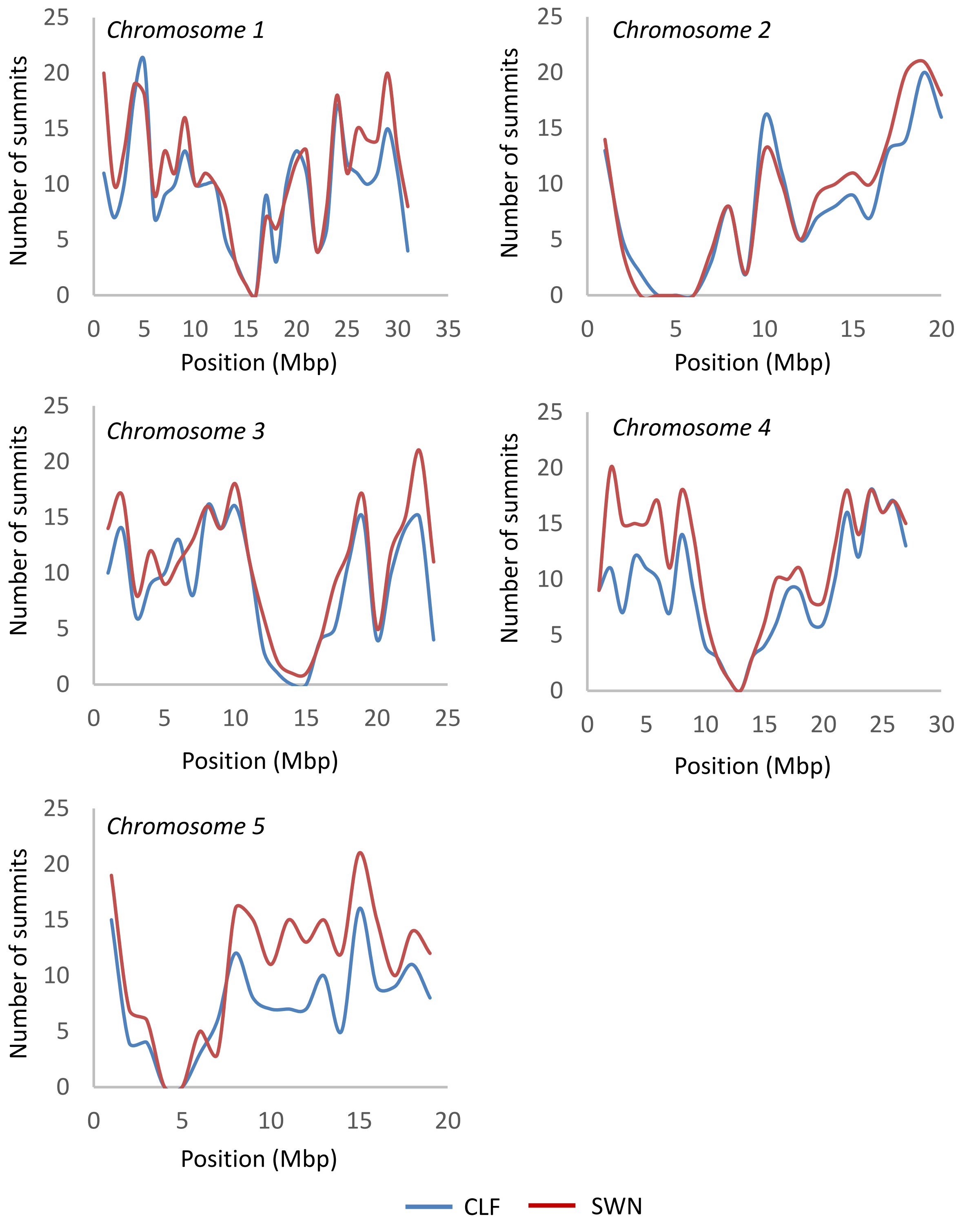

Figure S2. Number of the CLF and SWN binding summits in 1-Mbp windows along each of the chromosomes.

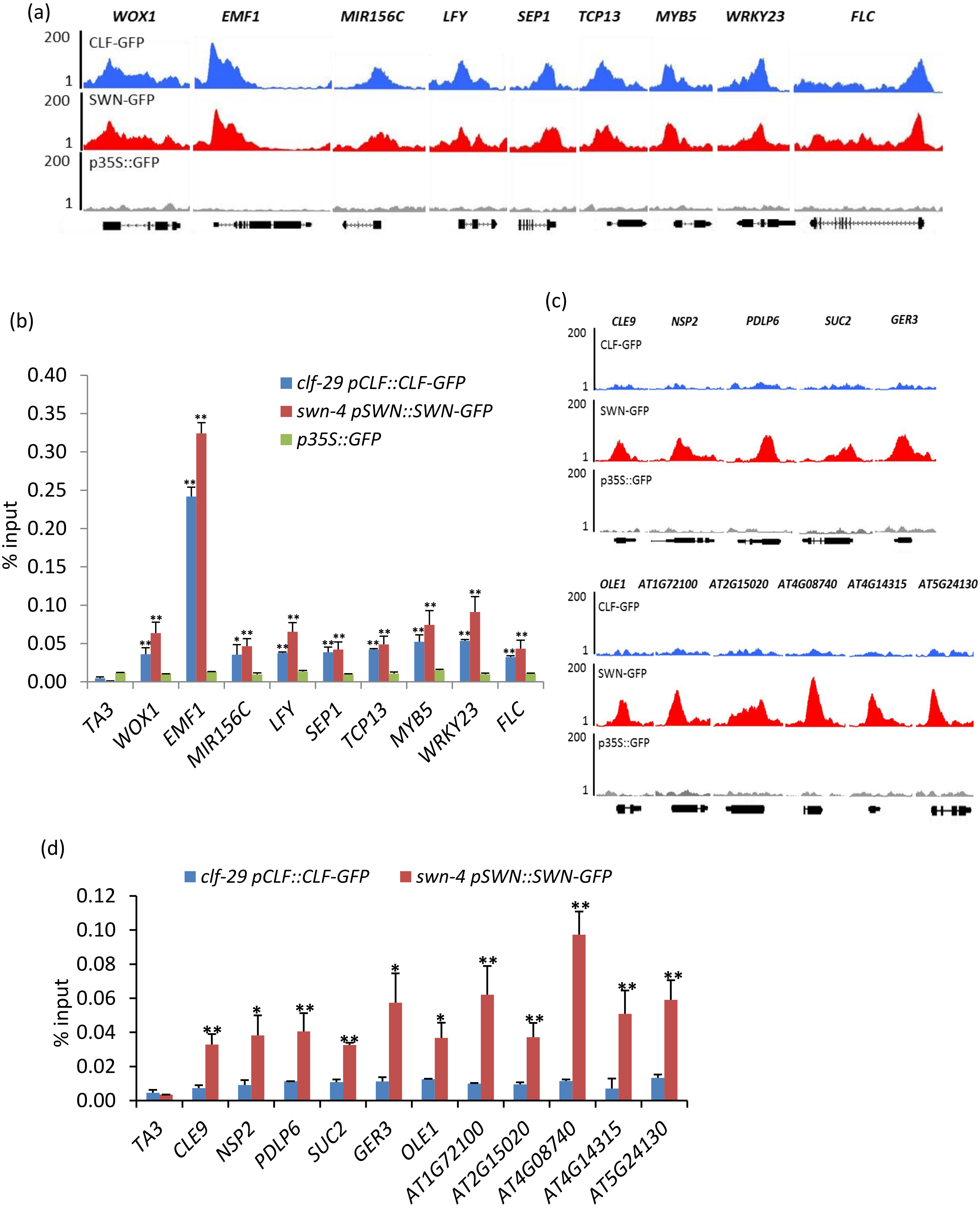

(a)
WOX1
EMF1
MIR156C
LFY
SEP1
TCP13
MYB5
WRKY23
FLC
CLF-GFP
SWN-GFP
p35S::GFP
(b)
clf-29 pCLF::CLF-GFP
swn-4 pSWN::SWN-GFP
p35S::GFP
% input
TA3
(c)
CLE9
NSP2
PDLP6
SUC2
GER3
OLE1
AT1G72100
AT2G15020
AT4G08740
AT4G14315
AT5G24130
(d)

Fig S3

Figure S3. ChIP-seq signals at selected loci occupied by CLF and/or SWN. (a, c) ChIP-seq genome browser views of CLF and SWN co-occupancy (a) and SWN unique occupancy (c) at selected genes. Gene structures are shown underneath the panel. (b, d) ChIP-qPCR validation for a and c, respectively. *p35S::GFP* plants were used as the negative control. ChIP signals are shown as percentage of input. *TA3*, a transposable element gene, was used as a negative control locus. Error bars indicate standard deviations among three biological replicates (Student's *t*-test, **p*<0.05, ***p*<0.01).

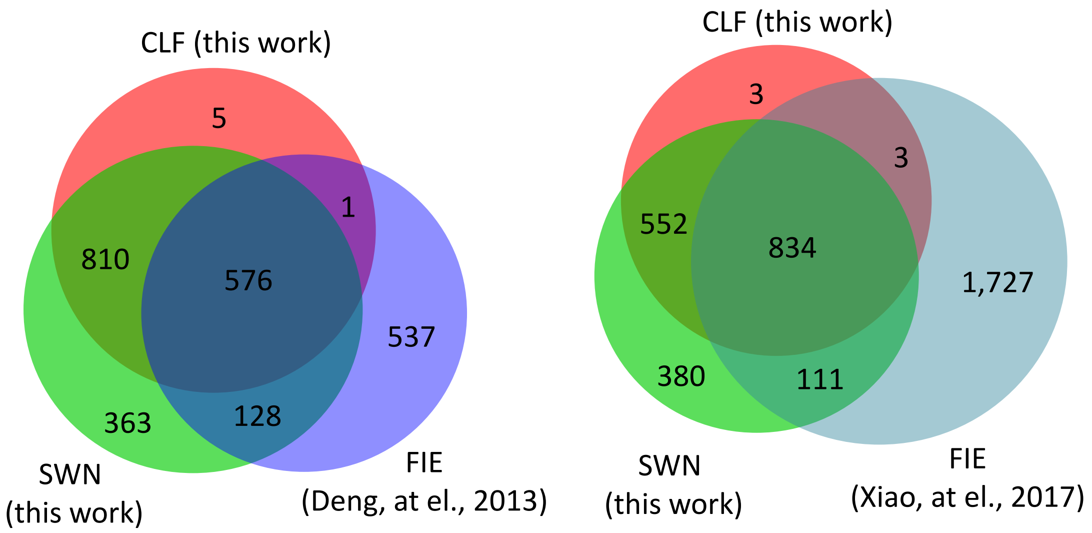

Figure S4. The overlap between CLF/SWN and FIE targets.

(a)

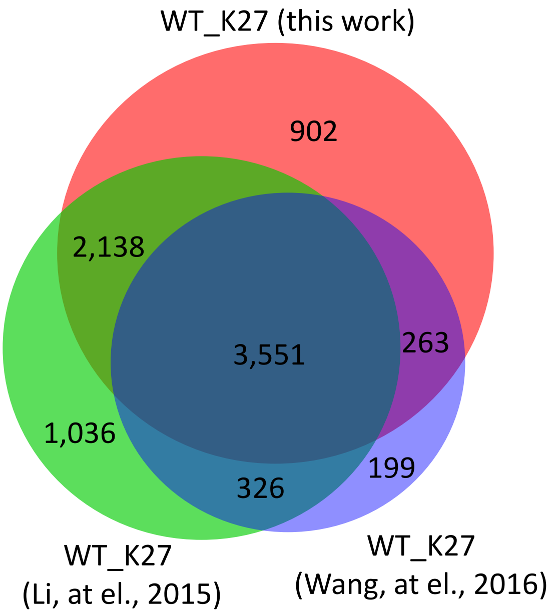

(b)

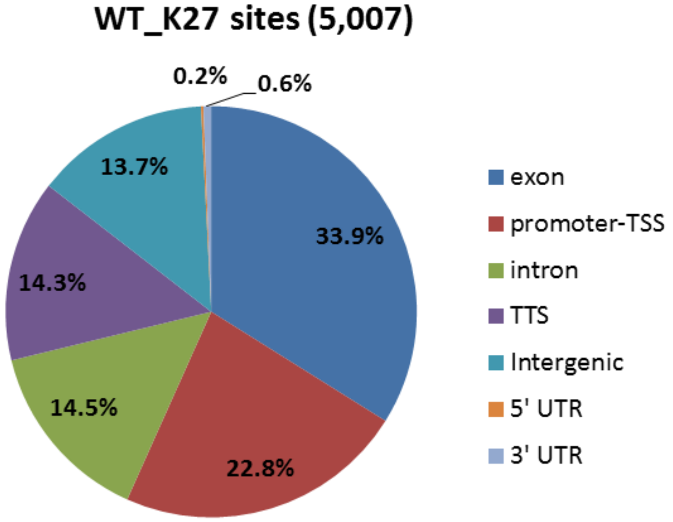

Figure S5. The distribution of H3K27me3 in *Arabidopsis* genome. (a) Comparison of H3K27me3 ChIP-seq (WT_K27) in this study with two recently published ChIP-seq data (Li et al., 2015; Wang et al., 2016). (b) Pie chart displaying the distribution of WT_K27 peaks at the annotated genic and intergenic regions in the genome. The analysis was done using a web-based analysis tool ChIPseek. WT, wild type.

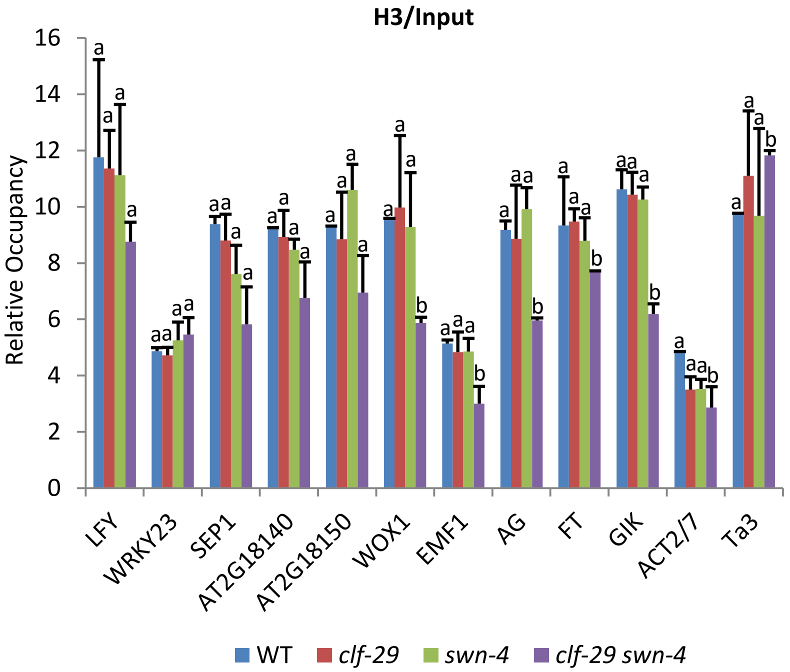

Figure S6. Histone H3 levels in WT, *clf-29*, *swn-4*, and *clf-29 swn-4*. Mean ± SD; three biological replicates were included; Lowercase letters indicate significant differences between genetic backgrounds, one-way ANOVA. WT, wild type.

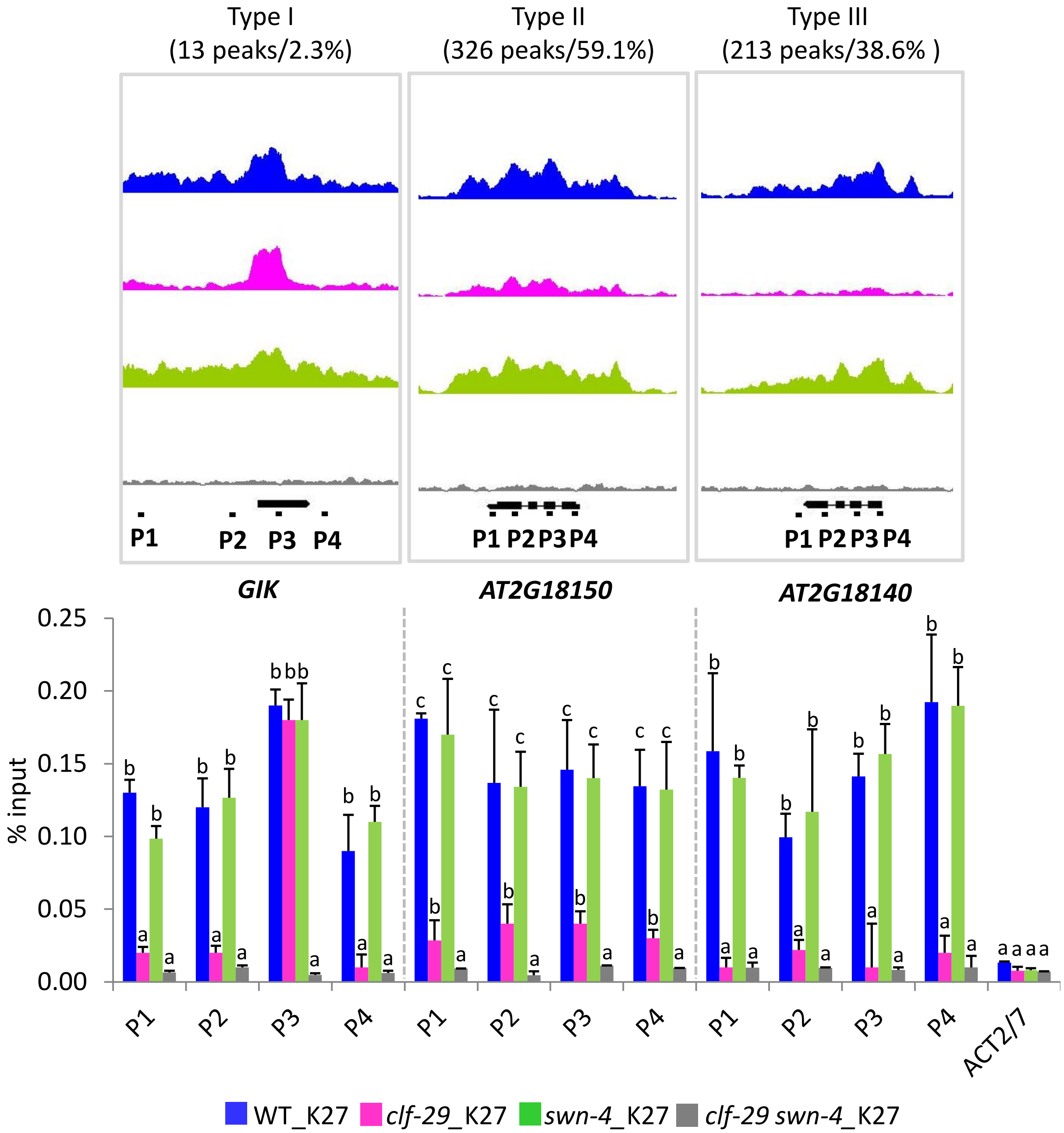

Figure S7. ChIP-qPCR validation of the ChIP-seq data illustrating three distinct types of reduction pattern of H3K27me3 levels in *clf-29*. Top: ChIP-seq signals at selected genomic loci showing three distinct types of H3K27me3 reduction pattern in *clf-29*. Gene structures are shown underneath the panel. Note that the locations of PCR primers used for ChIP-qPCR analysis presented below are indicated. Bottom: ChIP-qPCR validation results. ChIP signals are shown as percentage of input. *ACT2/7* was used as a negative control locus. Error bars indicate standard deviations among three biological replicates. Statistically significant differences are indicated by different lowercase letters, one-way ANOVA. WT, wild type.

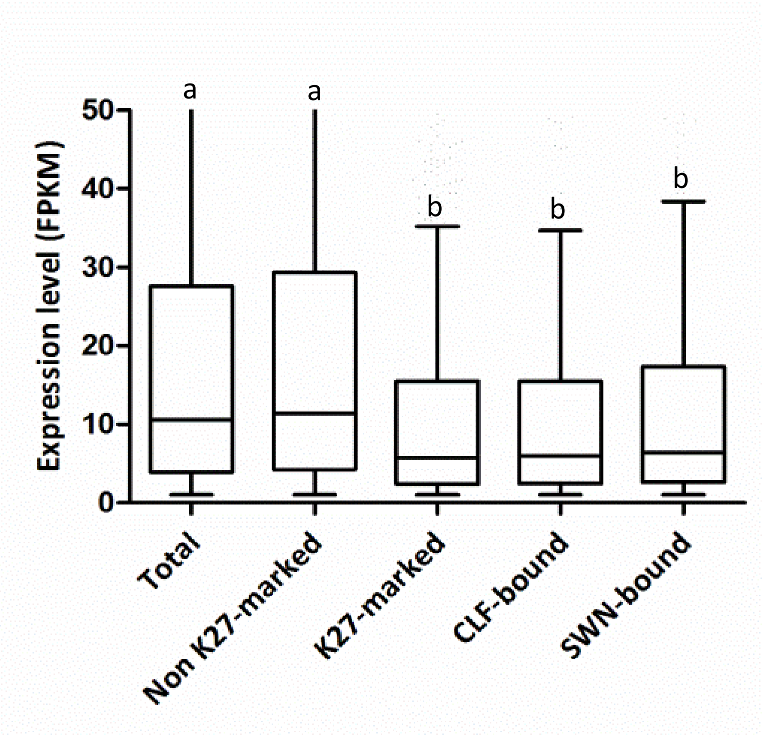

Figure S8. Box plot showing the average expression levels (FPKM) of the entire gene set in WT (total), and the subset of genes that are not marked by H3K27me3 (non K27-marked), marked by H3K27me3 (K27-marked), occupied by CLF (CLF-bound), or occupied by SWN (SWN-bound). Lowercase letters indicate significant differences among genetic backgrounds, one-way ANOVA. FPKM, fragments per kilobase per million. WT, wild type.

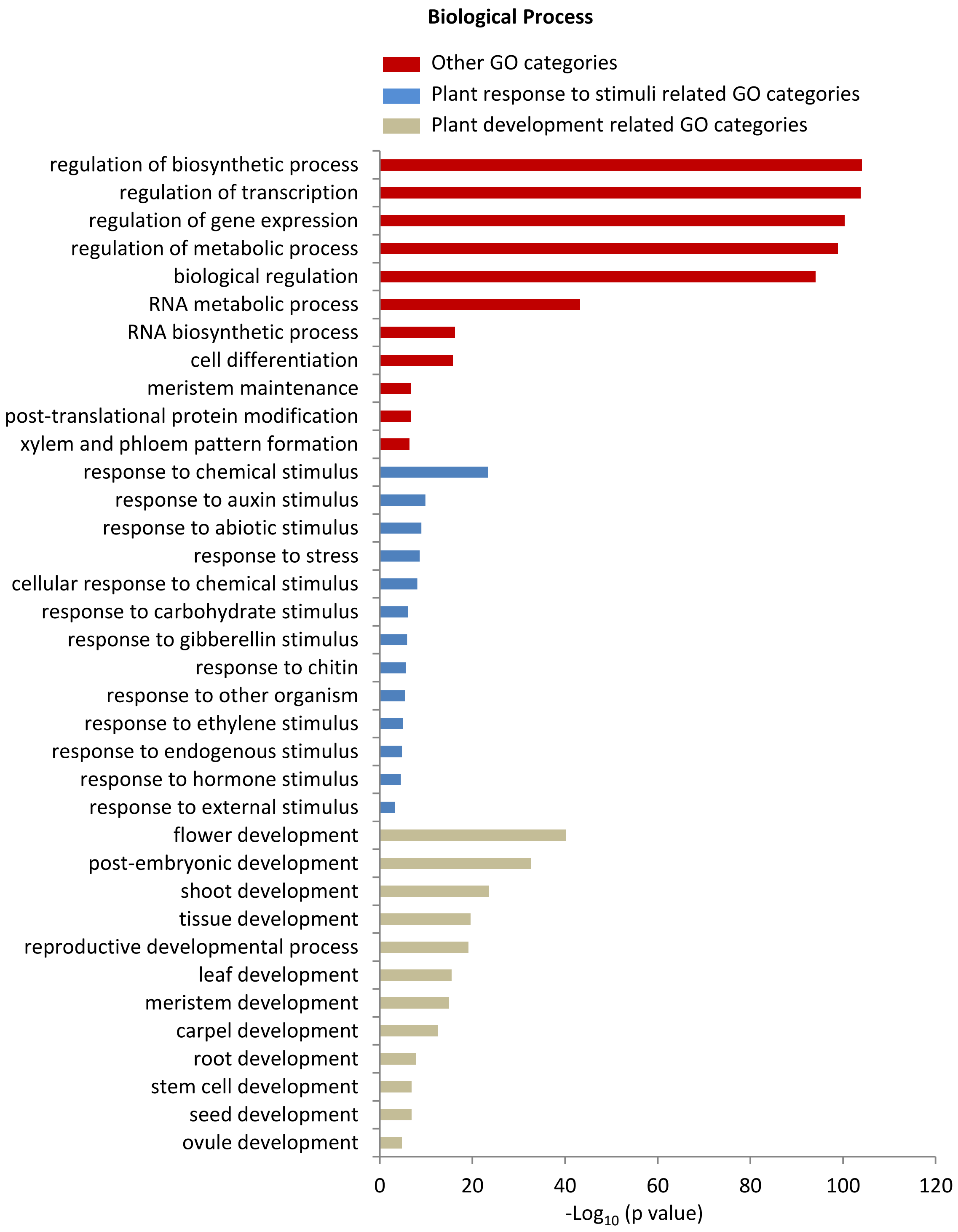

Figure S9. Gene Ontology (GO) analysis of CLF and SWN co-target genes showing up-regulation in *clf-29 swn-4* compared to WT using the AgriGO toolkit. WT, wild type.

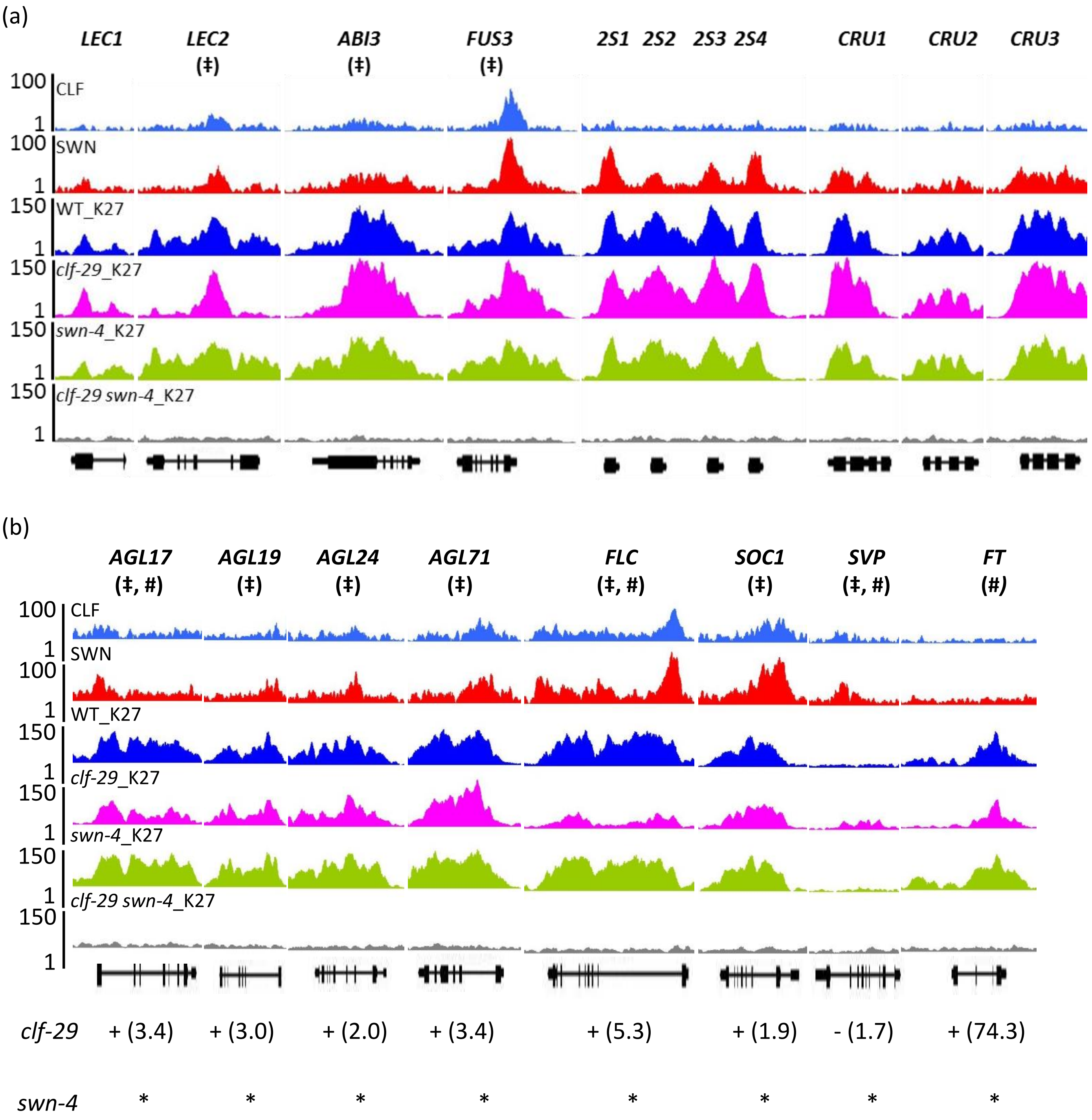

Figure S10. CLF/SWN and H3K27me3 ChIP-seq signals at some genes related to seed maturation and flowering. (a, b) ChIP-seq genome browser views of CLF and SWN occupancy as well as H3K27me3 (K27) levels in WT, *clf-29*, *swn-4*, and *clf-29 swn-4* at seed maturation regulator genes and seed storage protein genes (a); and major flowering transition genes (b). Gene structures are shown underneath each panel. B, Expression change at selected genes in *clf-29* and *swn-4* compared with those in WT. "+", "-", and "*" indicate up-regulation, down-regulation, and non-change, respectively. Numbers in brackets indicate the fold changes compared to WT. "‡" indicates genes bound by CLF, "#" represents reduction of K27 level in *clf-29*. WT, wild type.
