# Supplementary material for: Genome‐wide occupancy of histone H3K27 methyltransferases CURLY LEAF and SWINGER in Arabidopsis seedlings
Source: Plant Direct. 2019 Jan 31;3(1):e00100. doi: 10.1002/pld3.100 (PMC6508855; doi:10.1002/pld3.100)
Supplement: Supplementary file 2 [file PLD3-3-e00100-s002.pdf]

Table S1.

The overlap between CLF and SWN peaks

Sample \ Percent of overlap	Overlapped peak (> 50%)	Overlapped peak (> 75%)	Overlapped peak (=100%)
CLF/SWN peaks	952/91.5%	892/85.7%	398/38.2%

Table S2.

The DNA motifs in CLF and SWN peaks

Motifs \ Peaks	CLF		SWN	
	All (1,041)	Promoter-TSS (243)	All (1,298)	Promoter-TSS (311)
GAGA-like	24.6% (256)	60.5% (147)	25.6% (332)	36.3% (113)
Telo-box-like	9.2% (96)	25.5% (62)	6.5% (84)	19.0% (59)

Table S3.

List of CLF and SWN co-targets in different transcription factor (TF) families

TF families	Gene Lists
C2H2	AT1G03840, AT1G08290, AT1G10480, AT1G13290, AT1G14580, AT1G25250, AT1G55110, AT1G68130, AT1G68360, AT1G68480, AT2G01940, AT2G02070, AT2G02080, AT2G24500, AT2G29660, AT2G37740, AT3G13810, AT3G23130, AT3G45260, AT3G46070, AT3G46080, AT3G46090, AT3G51060, AT3G57670, AT3G58070, AT4G02670, AT4G17810, AT4G36260, AT5G01310, AT5G03150, AT5G06650, AT5G14010, AT5G22890, AT5G44160, AT5G66350
bHLH	AT1G02340, AT1G18400, AT1G22490, AT1G61660, AT1G68810, AT1G72210, AT1G74500, AT2G22750, AT2G22760, AT2G24260, AT2G34820, AT2G41130, AT2G43060, AT3G06120, AT3G06590, AT3G20640, AT3G25710, AT3G50330, AT3G56770, AT4G00120, AT4G00870, AT4G25400, AT4G25410, AT4G29100, AT4G30410, AT4G30980, AT4G37850, AT5G09750, AT5G15160, AT5G39860, AT5G46830, AT5G51780, AT5G51790, AT5G53210, AT5G56960, AT5G67060
AP2-EREBP	AT1G03800, AT1G12630, AT1G12980, AT1G15360, AT1G21910, AT1G24590, AT1G28360, AT1G33760, AT1G51190, AT1G63030, AT1G72570, AT1G75490, AT1G77200, AT1G80580, AT2G31230, AT2G33710, AT2G35700, AT2G39250, AT2G40220, AT2G47520, AT3G20840, AT3G23230, AT3G60490, AT5G10510, AT5G11190, AT5G17430, AT5G18560, AT5G57390
MYB	AT1G17950, AT1G18570, AT1G18710, AT1G26780, AT1G73410, AT3G13540, AT3G13890, AT3G23250, AT3G27810, AT3G62610, AT4G21440, AT5G10280, AT5G12870, AT5G17800, AT5G23000, AT5G40350, AT5G57620, AT5G58850, AT5G61420, AT5G62470
MADS	AT1G17310, AT1G24260, AT1G69120, AT1G77950, AT2G03710, AT2G14210, AT2G22540, AT2G22630, AT2G42830, AT2G45660, AT3G02310, AT3G54340, AT3G58780, AT4G09960, AT4G18960, AT4G22950, AT4G24540, AT5G10140, AT5G13790, AT5G15800, AT5G20240, AT5G23260, AT5G49490, AT5G51860, AT5G51870, AT5G58890, AT5G60910, AT5G62165
Homeobox	AT1G05230, AT1G23380, AT1G62360, AT1G62990, AT1G79840, AT2G17950, AT2G22800, AT2G23760, AT2G27990, AT2G28610, AT2G32370, AT2G44910, AT3G03660, AT3G18010, AT3G60390, AT3G61150, AT4G00730, AT4G04890, AT4G08150, AT4G17460, AT4G21750, AT4G36870, AT5G02030, AT5G03790, AT5G06710, AT5G15150, AT5G41410, AT5G45980, AT5G46880, AT5G59340, AT5G66700
WRKY	AT5G49520, AT1G29280, AT1G30650, AT1G62300, AT1G69310, AT1G69810, AT1G80840, AT2G21900, AT2G44745, AT2G47260, AT3G56400, AT4G01720, AT4G04450, AT4G18170, AT4G22070, AT4G23550, AT4G39410, AT5G01900, AT5G46350, AT5G52830
TCP	AT1G69690, AT3G02150, AT3G18550, AT3G47620, AT4G18390, AT5G08070, AT5G60970
NAC	AT1G12260, AT1G52890, AT1G54330, AT1G62700, AT1G65910, AT1G71930, AT1G79580, AT2G02450, AT2G18060, AT2G46770, AT3G04060, AT3G15500, AT3G15510, AT3G18400, AT4G28530, AT4G36160, AT5G53950, AT5G56620, AT5G66300

Table S4.

Oligonucleotides used in this study

Primer	Sequence (5' to 3')	Application
SWN-Pme1-F	AGCTTTGTTTAAACTCACTTATCTACTCGTGTTATGGGCAC	Cloning
SWN-Asc1-R	TTGGCGCGCCAATGAGATTGGTGCTTTCTGGCTCTACG	Cloning
CLF-Pme1-F	AGCTTTGTTTAAACTTGTCGATAGTCAAGGAATTGTACCTC	Cloning
CLF-Asc1-R	TTGGCGCGCCAAGCAAGCTTCTTGGGTCTACCAACAG	Cloning
clf-29-RP	GAGGCATTGACTTTGATTTGC	Genotyping
clf-29-LP	AAGAAACTTGCTAGTTCCGCC	Genotyping
swn-4-RP	CGAGGAATTTTCTAATTCCGG	Genotyping
swn-4-LP	TGATTATTGCTCCGTTTCCAC	Genotyping
LBb1	GCGTGGACCGCTTGCTGCAACT	Genotyping
CLF-1-F	AAGCATTTACCTGGGGGTCG	Sequencing
CLF-1-R	GAGCCCGGGTATGATTCTGG	Sequencing
CLF-2-F	CCAGCAACGTTCGTGTTTGT	Sequencing
CLF-2-R	ACCATTCCTGAACTGGCAGG	Sequencing
CLF-3-F	GAGGCCATTGACGACGAAGA	Sequencing
CLF-3-R	TGGAGAACTTCGTGGGTGTG	Sequencing
CLF-4-F	GCTGAGCGAGTTCCTCGTAA	Sequencing
CLF-4-R	TGCAGCTCTTTGGGCAACTA	Sequencing
CLF-5-F	AGCCCTGCCGTCAGTTTAAT	Sequencing
CLF-5-R	CACCCTGTGATCTCCAGCAA	Sequencing
CLF-R-4693	ACTTTCATCACTACCCGGCG	Sequencing
CLF-F-2675	GAGGTCTTCTATGGAACGAGGAGG	Sequencing
CLF-F-6144	GGAAGATATACGATCGCGAG	Sequencing
SWN-1-F	TGCAATGACGGGACACCTAC	Sequencing
SWN-1-R	TGAGCACGCCCTGAACTAAG	Sequencing
SWN-2-F	CTTAGTTCAGGGCGTGCTCA	Sequencing
SWN-2-R	GTTGCTATCGTCCGTCACCA	Sequencing
SWN-3-F	CTCCAGAGCCAGGCATGTCG	Sequencing
SWN-3-R	TGTCTTCGACCAACCACAGAC	Sequencing
SWN-4-F	TGCTGAGAGAATACCGCCATAC	Sequencing
SWN-4-R	TGTTTGCTGCAGGGTTTCCT	Sequencing
SWN-5-F	GGCGGTCAGAGAAGTACCAG	Sequencing
SWN-5-R	CAACAATGCCCAAAGTCGCA	Sequencing
SWN-6-F	TGGTGGCAAAAACCAGTCCT	Sequencing
SWN-6-R	CCTTTGCGTTGAGCATCGAG	Sequencing
SWN-F-985	GTACAGGTGATTAGTTTGTACTG	Sequencing
SWN-F-3973	ACTGTCGTCTGCATGGATGTT	Sequencing
SWN-F-5199	GATCTAGTACCCCAAATTTG	Sequencing
SWN-F-6790	GAAGGAGGACTAGGATTATAAC	Sequencing
SWN-R-7050	TATGCGGGAAGCGATGAGAC	Sequencing

Ta3-F	GATTCTTACTGTAAAGAACATGGCATTGAGAGA	qChIP-PCR
Ta3-R	TCCAAATTTCCTGAGGTGCTTGTAACC	qChIP-PCR
WOX1-F	CATCACCATTTCGATGACAAG	qChIP-PCR
WOX1-R	CGTAACCTCATTCTCAATTCACC	qChIP-PCR
EMF1-F	TTCTCTCTCCCTCCCCTTTC	qChIP-PCR
EMF1-R	AGCGGCAAGGGATAAAAACT	qChIP-PCR
MIR156C-F	TGTTTGGACCAGCCTAAGAAA	qChIP-PCR
MIR156C-R	GATTTGGTTCCCAATTGCAT	qChIP-PCR
LFY-F	ATTGGTTCAAGCACCACCTC	qChIP-PCR
LFY-R	TCAAGCTCCTCGTCCTTCAT	qChIP-PCR
SEP1-F	CTTTCCACGGTTGGAGAAGA	qChIP-PCR
SEP1-R	GAGGAAGAGTAGAGCTGAAGAGGA	qChIP-PCR
TCP13-F	CGCTACGACAAGACGTGAAA	qChIP-PCR
TCP13-R	CGCGTGAAACCCTAACAATC	qChIP-PCR
MYB5-F	GGGGATGAAGAGAGGACCAT	qChIP-PCR
MYB5-R	CGACGTAATTCCTCCACGTT	qChIP-PCR
WRKY23-F	ACTACCCGTCGTCACAAAGC	qChIP-PCR
WRKY23-R	CAAAGTCTTGATGCTGCTGAG	qChIP-PCR
FLC-F	CCTAATTTGATCCTCAGGTTTGGG	qChIP-PCR
FLC-R	CCGACGAAGAAAAAGTAGATAGGCA	qChIP-PCR
CLE9-F	ATCCTCCACCGCTTCTTCAA	qChIP-PCR
CLE9-R	TGAGGAGTCACGTGGTAAGG	qChIP-PCR
NSP2-F	GCTTCTCCCACTTCAACGTC	qChIP-PCR
NSP2-R	GACCCAAAAGCTCACGAAGG	qChIP-PCR
PDLP6-F	CACCACCGAGTCTCTACGAG	qChIP-PCR
PDLP6-R	TGAGGATAAGACGGTGGTGG	qChIP-PCR
SUC2-F	TCCGGCTGATCTAACTCACC	qChIP-PCR
SUC2-R	CACTACAACCACCGCAACAA	qChIP-PCR
GER3-F	GGCCTTTCCCAGAGTTTAGC	qChIP-PCR
GER3-R	TTTGCGCCGGATTTATCTCC	qChIP-PCR
OLE1-F	TCATCGGCAGAGACCAGTAC	qChIP-PCR
OLE1-R	GTAGTCAGATCCTCGTCCGG	qChIP-PCR
AT1G72100-F	CTTTACTAGCGGCGGAGGTA	qChIP-PCR
AT1G72100-R	ATGACTTCTCCCTCTGACGC	qChIP-PCR
AT2G15020-F	AGTCCGTTCGGCTCTAAGTT	qChIP-PCR
AT2G15020-R	CGAGTCTGATTCACACGCAG	qChIP-PCR
AT4G08740-F	GCTCATCAACTCGTCATGCA	qChIP-PCR
AT4G08740-R	TGAGCTTCGTGTCCAAGGAT	qChIP-PCR
AT4G14315-F	TCGGTTTTGGCGATTGTTGT	qChIP-PCR
AT4G14315-R	TGGAAGAGATGGTGCAGTCA	qChIP-PCR
AT5G24130-F	AGCAGAGCCGAGTACATCAA	qChIP-PCR
AT5G24130-R	TGAGAATCCTGTCGCTGTGA	qChIP-PCR
GIK-1-F	GTATTTGTTTAAGTTCTAACTAAG	qChIP-PCR

GIK-1-R	GGGAAGAAAACGCGGAGATC	qChIP-PCR
GIK-2-F	GACTTAACCATTGGAGTAC	qChIP-PCR
GIK-2-R	GAAAGTATTAACTAGATCTG	qChIP-PCR
GIK-3-F	ACTCTTAGGGCTCACATTC	qChIP-PCR
GIK-3-R	CTGACGGATGCTGACGTTAG	qChIP-PCR
GIK-4-F	CCTCTAGAAAAAAGAGCTTG	qChIP-PCR
GIK-4-R	CCACAATAGGCAAGTTCATTC	qChIP-PCR
AT2G18150-1-F	GCAAGTTAGCAGCGTAGGAT	qChIP-PCR
AT2G18150-1-R	CAGAGACTGTACAACCAATCCG	qChIP-PCR
AT2G18150-2-F	GGTGAGATCGAGACCTTGGT	qChIP-PCR
AT2G18150-2-R	CATTCCTGCACCCAACAACA	qChIP-PCR
AT2G18150-3-F	AACAACTTCAAACCCGCGAG	qChIP-PCR
AT2G18150-3-R	GGTTGTGATGGATCGTTGCT	qChIP-PCR
AT2G18140-4-F	GGCTACAACTGACCTCACGA	qChIP-PCR
AT2G18150-4-R	GAGAGTAACTATGGCGGCGA	qChIP-PCR
AT2G18140-1-F	TCTCACCACTCGAACCTGTC	qChIP-PCR
AT2G18140-1-R	GGGACTGTTGAATTCCGACC	qChIP-PCR
AT2G18140-2-F	GTCGAACAAGTTGTCGGGTT	qChIP-PCR
AT2G18140-2-R	GGAAGAAGAGATTCGGCAACT	qChIP-PCR
AT2G18140-3-F	ACAAGAGACAGTGTTAGGGCA	qChIP-PCR
AT2G18140-3-R	CTCGCGGGTTTGAAGTTGTT	qChIP-PCR
AT2G18140-4-F	CACGATCTCCTCCGCTCTAG	qChIP-PCR
AT2G18140-4-R	GAGTAACTTTGGCGGCAACA	qChIP-PCR
AG-F	TCGGAGCTAGGAGGAGATTC	qChIP-PCR
AG-R	CGATTCGTTGTGTTCTCGAT	qChIP-PCR
FT-F	CCAGATGTTCCAAGTCCTAGCAACC	qChIP-PCR
FT-R	GGTGTGGGCTTTTTTGGGAGAC	qChIP-PCR
ACT2/7-F	CGTTTCGCTTTCCTTAGTGTTAGCT	qChIP-PCR
ACT2/7-R	AGCGAACGGATCTAGAGACTCACCTTG	qChIP-PCR

Table S5.

The ChIP-seq reads number of all the experiments in this study

Samples	Total reads	Uniquely mapped reads	% uniquely mapped
CLF-GFP Rep1	13,000,000	6,700,000	52
CLF-GFP Rep2	12,000,000	6,400,000	53
SWN-GFP Rep1	42,000,000	9,500,000	23
SWN-GFP Rep2	41,000,000	13,000,000	32
WT_K27 Rep1	14,000,000	11,000,000	79
WT_K27 Rep2	14,000,000	11,000,000	79
*clf-29*_K27 Rep1	15,000,000	11,000,000	73
*clf-29*_K27 Rep2	14,000,000	11,000,000	79
*swn-4*_K27 Rep1	16,000,000	11,000,000	69
*swn-4*_K27 Rep2	12,000,000	10,000,000	83
*clf-29 swn-3*_K27 Rep1	11,000,000	6,200,000	56
*clf-29 swn-3*_K27 Rep2	14,000,000	8,500,000	61

Table S6.

The RNA-seq reads number of all the experiments in this study

Samples		Total reads	Mapped reads	Aligned pairs of
WT Rep1	Forward	24,247,121	23,569,958	21,545,227
	Reverse	24,247,121	21,898,473	
WT Rep2	Forward	23,453,341	22,619,791	19,878,956
	Reverse	23,453,341	20,295,303	
WT Rep3	Forward	17,662,886	17,062,223	15,649,123
	Reverse	17,662,886	15,937,038	
clf-29 Rep1	Forward	20,601,270	18,797,720	18,143,120
	Reverse	20,601,270	18,698,512	
clf-29 Rep2	Forward	18,695,240	18,090,129	16,798,857
	Reverse	18,695,240	17,106,009	
clf-29 Rep3	Forward	22,425,331	21,786,431	20,009,345
	Reverse	22,425,331	20,353,879	
swn-4 Rep1	Forward	18,630,733	18,008,285	16,637,370
	Reverse	18,630,733	16,952,099	
swn-4 Rep2	Forward	24,677,440	23,894,738	22,779,345
	Reverse	24,677,440	23,208,147	
swn-4 Rep3	Forward	24,154,138	23,493,065	21,191,193
	Reverse	24,154,138	21,545,710	
clf-29 swn-4 Rep1	Forward	23,896,240	22,776,814	21,859,458
	Reverse	23,896,240	22,445,589	
clf-29 swn-4 Rep2	Forward	23,065,073	22,008,176	21,205,347
	Reverse	23,065,073	21,817,292	
clf-29 swn-4 Rep3	Forward	25,944,577	24,735,884	23,818,219
	Reverse	25,944,577	24,492,594	
