# Supplementary material for: Genome‐wide occupancy of histone H3K27 methyltransferases CURLY LEAF and SWINGER in Arabidopsis seedlings
Source: Plant Direct. 2019 Jan 31;3(1):e00100. doi: 10.1002/pld3.100 (PMC6508855; doi:10.1002/pld3.100)
Supplement: Supplementary file 13 [file PLD3-3-e00100-s013.pdf]

Response to reviewers' comments

We are grateful to the three reviewers for their prompt evaluation of our submission and insightful comments and suggestions. Our point-to-point responses are presented below (our answers are in dark blue).

Reviewer: 1

This manuscript addresses the interesting question about the redundancy of E(z) proteins in mediating H3K27me3-dependent gene silencing in plants (Arabidopsis thaliana). The question is addressed on a genome-wide level by combining ChIP-seq and RNA-seq approaches and focuses on the potential role of CLF and SWN in Arabidopsis thaliana. These original data are also compared to published data.
The main novel findings are the genome-wide occupancy of CLF and SWN and the possibility that these two proteins may largely co-occupy the same target genes. This last point remains speculative given that the ChIP-seq data were obtained from whole seedlings and that no sequential ChIP experiments were performed. Additional correlative analyses mainly confirm previous conclusions drawn from FIE or LHP1 ChIP-seq and from the numerous H3K27me3 profiling performed in Arabidopsis.

The manuscript is generally well written and the data appear sound and convincing. Whereas the work presented could represent an interesting advance in the understanding of PRC2 activity in plants, it falls short of this objective as it provides limited novelty on this topic.

Answer: Thanks for the positive comments. We agree that the main novel finding from this study is the genome-wide occupancy of CLF and SWN and the possibility that these two proteins may largely co-occupy the same target genes and functionally interplay. Although PRC2, as well as the H3K27me3 mark, has been intensively studied, a single combined, comprehensive study/analysis of their functions is still lacking. Therefore, we focused on the genome-wide occupancy of CLF/SWN binding and H3K27me3 distribution, together with the transcriptome data in each mutant background, aiming to provide a more detailed profiling of PRC2 and H3K27me3. All these data generated from this study will be useful resources for the plant epigenetics research community to decipher the hidden details of PRC2 activities, targeting mechanisms, and their roles in growth and development. The sequential ChIP-re-ChIP experiment is a very good suggestion. We will design and perform the experiment in the near future, but it would not be the focus of this work.

Additional points:
- Do the translational fusions generated complement the double mutant clf swn?
Answer: Yes, the translational fusions (CLF and SWN) generated can complement *clf swn*. We have crossed the CLF and SWN fusions with the *clf swn* double mutant, respectively, and both of them could rescue the callus-like phenotype of *clf swn*.

- It is not clear why the confocal images presented in Fig1C are not from the same plane.
Answer: Thanks for the comment. We have replaced the confocal image presented in Figure 1c.

- The only partial overlap between CLF/SWN and FIE (or H3K27me3) is unlikely to be entirely explained by technical reasons or by the fact that the experiments were performed in different tissues or with plants of different ages. In addition to the points raised in the discussion, the authors would be inspired to refer to the literature in mammals and drosophila, as such partial overlaps were shown to be indicative of the respective functions of the distinct PRC2 subunits.

Answer: Thanks for the insightful comment. We agree that, in addition to the technical reasons, the partial overlap between CLF/SWN and FIE might also imply that CLF/SWN and FIE probably have distinct functions, although the *clf swn* double mutant shares similar phenotypes with the *fie* mutant (Chanvivattana et al., 2004, Bouyer et al., 2011). The partial overlap between H3K27 methyltransferase EZH1 and EED has been reported in mammals (Bodega et al., 2017). We have added this point in the Discussion. As we indicated in the Discussion, one likely explanation for the smaller number of CLF and SWN peaks compared to the H3K27me3 peaks could be that CLF and SWN bind to chromatins transiently, they might leave target sites as long as the H3K27me3 mark is deposited. We also need to bear in mind that ChIP-seq profiling is just a snapshot of the occupancy, but the H3K27me3 mark observed is the culmination of PRC2 activity at all earlier stages, events that cannot be fully recorded at just one time point.

Reviewer: 2

This manuscript reports on a ChIP-seq analysis of the PRC2 H3K27 methyltransferases CLF and SWN binding sites across the genome of Arabidopsis. The authors also carry out analysis of H3K27me3 distribution and transcriptome analysis in the clf swn double mutant with which to compare the CLF and SWN binding sites. The paper is well-written and easy to follow. The methods and analysis tools employed are standard for these types of analyses and hence the data appear to be sound. The analysis and interpretation of the data is reasoned, I did not find any major flaws.

The authors show that CLF and SWN share similar sets of binding sites, consistent with their largely redundant function based on mutant analysis. The sites correspond to H3K27me3 peaks and contain DNA motifs that have also been associated with binding sites of other PRC2 components.

The paper largely confirms work that has been published over the last 5 or so years on the distribution and nature of PRC2 binding sites in the Arabidopsis genome.

Answer: Thanks for the positive comments.

Reviewer: 3

The paper presents whole genome profiling of the Polycomb group proteins CLF and SWN, the epigenetic mark that they catalyse (H3K27me3), and the effects of single and double mutants on H3K27me3 levels and transcription. The data confirms the redundancy between CLF and SWN observed in other studies and also reveals that mutating SWN has little effect on H3K27me3 levels or target gene expression. Strikingly, although most CLF and SWN targets are marked with H3K27me3, most H3K27me3 genes are not marked with CLF or SWN, although loss of CLF and SWN activity removes H3K27me3 genome wide. This suggests that the detection of CLF/SWN binding is less sensitive, i.e. only the more strongly bound targets are found, but those that are found are likely to be real. Motif analysis confirms the association of PcG targets with GAGA and telobox motifs, as has been described in several recent studies.

Overall the data seems very consistent with findings from previous studies and of good technical quality. A possible criticism is that it doesn't provide much mechanistic or novel insights. However, much of the data is either novel (SWN binding, H3K27me3 in swn mutants etc) or has not been previously combined

in a single coherent study. I therefore agree with the authors that the study will be a useful resource for the plant epigenetics community.

Answer: Thanks for the positive comments.

I have few comments, as follows:

1. It would be useful to plot CLF and SWN binding along chromosomes, e.g. to confirm whether binding is predominantly euchromatic and low in pericentromeric regions, as has been found for e.g H3K27me3.
Answer: Thanks for the great suggestion. The plots of CLF and SWN binding along chromosomes have been included in Figure S2. The CLF/SWN binding is predominantly in euchromatic regions.

2. In discussing the potential role of CLF in H3K27me3 spreading, the recent Science paper from Hongchun Yang and Caroline Dean and others should be cited as this describes a role for CLF in spreading of H3K27me3 after vernalization treatment.
Answer: As suggested, the papers which describe a role for CLF in spreading of H3K27me3 after vernalization treatment have been cited in the Discussion section.

3. Supplementary Table S2 lists genes uniquely bound by SWN but not those uniquely bound by CLF. Admittedly there are only 6, but for consistency this column should be included.
Answer: Thanks for the suggestion. We have added a column showing the genes uniquely bound by CLF in Data S2.

4. Venn diagram is repeatedly referred to as Veen diagram
Answer: The correction has been made through out the whole manuscript. Thanks.

5. In Figure 4E I have the impression that H3K27me3 levels at CLF/SWN targets are slightly elevated in swn mutants relative to wild type. Supplemental data set S7 indicates that at the level of two fold change relatively few genes show increase in swn mutant backgrounds, but I wondered if at lower cut offs there was a trend for increase.
Answer: There was no clear trend for increase of H3K27me3 in *swn* compared to WT when lower cut offs (1.8-fold and 1.5-fold) were applied. As shown in Figure 4d and 4e, the genome-wide H3K27me3 level in *swn* is very similar to that in WT.

6. Although the majority of genes misexpressed in clf or clf swn mutants are not direct targets, I wondered if genes were binned according to fold change mis expression whether this would change at all – for example, if one took the top 10% of upregulated genes (ranked by fold change) would these include a higher percentage of CLF/SWN targets than the overall cohort?
Answer: We have re-analyzed the correlation between CLF/SWN binding and mis-expression in *clf swn*. As suggested, we took the top 10% of up-regulated genes (605 out of 6058), and found out that 139 genes were bound by CLF. The percentage (139/605=23.0%) was indeed much higher than the overall cohort (476/6058=8.0%). Thanks for the insightful comment.

7. Line 62 FIS2 gene name lacks spacing.
Answer: Thanks, it has been corrected.
